# Development and validation of a tumor immune cell infiltration-related gene signature for recurrence prediction by weighted gene co-expression network analysis in prostate cancer

**DOI:** 10.3389/fgene.2023.1067172

**Published:** 2023-03-16

**Authors:** Lin-Ying Xie, Han-Ying Huang, Yu-Lei Hao, Miaomiao Yu, Wenju Zhang, Enwei Wei, Chunfeng Gao, Chang Wang, Lei Zeng

**Affiliations:** ^1^ Bethune Institute of Epigenetic Medicine, The First Hospital of Jilin University, Changchun, Jilin, China; ^2^ International Center of Future Science, Jillin University, Changchun, Jilin, China; ^3^ State Key Laboratory of Oncology in South China, Collaborative Innovation Center for Cancer Medicine, Sun Yat-Sen University Cancer Center, Guangzhou, China; ^4^ Department of Neurology and Neuroscience Center, The First Hospital of Jilin University, Changchun, Jilin, China; ^5^ Cancer Center, The First Hospital of Jilin University, Changchun, Jilin, China

**Keywords:** prostate cancer, tumor microenvironment, tumor immune infiltrating cells, prognosis prediction, tumor mutation burden, clinical therapy, dendritic cells

## Abstract

**Introduction:** Prostate cancer (PCa) is the second most common malignancy in men. Despite multidisciplinary treatments, patients with PCa continue to experience poor prognoses and high rates of tumor recurrence. Recent studies have shown that tumor-infiltrating immune cells (TIICs) are associated with PCa tumorigenesis.

**Methods:** The Cancer Genome Atlas (TCGA) and Gene Expression Omnibus (GEO) datasets were used to derive multi-omics data for prostate adenocarcinoma (PRAD) samples. The CIBERSORT algorithm was used to calculate the landscape of TIICs. Weighted gene co-expression network analysis (WGCNA) was performed to determine the candidate module most significantly associated with TIICs. LASSO Cox regression was applied to screen a minimal set of genes and construct a TIIC-related prognostic gene signature for PCa. Then, 78 PCa samples with CIBERSORT output *p*-values of less than 0.05 were selected for analysis. WGCNA identified 13 modules, and the MEblue module with the most significant enrichment result was selected. A total of 1143 candidate genes were cross-examined between the MEblue module and active dendritic cell-related genes.

**Results:** According to LASSO Cox regression analysis, a risk model was constructed with six genes (STX4, UBE2S, EMC6, EMD, NUCB1 and GCAT), which exhibited strong correlations with clinicopathological variables, tumor microenvironment context, antitumor therapies, and tumor mutation burden (TMB) in TCGA-PRAD. Further validation showed that the UBE2S had the highest expression level among the six genes in five different PCa cell lines.

**Discussion:** In conclusion, our risk-score model contributes to better predicting PCa patient prognosis and understanding the underlying mechanisms of immune responses and antitumor therapies in PCa.

## 1 Introduction

Prostate cancer (PCa) is the second most prevalent malignancy (after lung cancer) in men (Sung et al., 2021). According to GLOBOCAN in 2020, 1.4 million new cases of prostate cancer have been globally reported, with 375,000 PCa-related deaths (Sung et al., 2021). However, the clinical characteristics, incidence, and mortality rates of PCa vary considerably worldwide, suggesting that there are differing genetic, environmental, and other underlying risk factors in different countries (Sung et al., 2021; Siegel et al., 2022). Both genetic mutations (Jin et al., 2019) and epigenetic alterations (Yang et al., 2021b) are molecular mechanisms underlying the occurrence and development of malignant tumors, including PCa. Currently, PCa treatment includes active surveillance (Jeong et al., 2020), surgery (Tilki et al., 2019), radiotherapy (Fillon, 2020), androgen deprivation therapy (ADT) (Guan et al., 2022), chemotherapy (Petrylak et al., 2004), targeted α-therapy (radium-223) (Parker et al., 2013), immunotherapy (Redman et al., 2018), and a combination of these therapies (Kishan et al., 2022). However, the appropriate selection of an therapeutic strategy can be involute and depends on several key factors, such as patient age, disease stage, functional status, metastasis, and response to previous therapies. Therefore, an accurate method for early risk stratification is necessary for accurately evaluating the prognosis, customizing patient-specific therapeutic interventions, and long-term management of PCa. Immune checkpoint inhibitors (ICIs) (anti-CTLA-4, anti-PD-1, anti-PD-L1, or their combinations) have produced remarkable responses and improved overall survival in certain types of advanced cancers, such as hematological, lung, bladder, and skin cancers (Kwon et al., 2014; Beer et al., 2017; Antonarakis et al., 2020; Powles et al., 2022). However, the majority of PCa patients do not respond to and thus benefit from current ICI treatments, and some even experience immune-related adverse effects. Therefore, it is urgent to elucidate the molecular mechanism of the tumor and look for a predictive signature, which will be beneficial to the diagnosis, prognosis prediction and ICI treatment for PCa atients.

The tumor microenvironment (TME) has been widely implicated in tumorigenesis. The TME is a highly complex and heterogenous ecosystem, where tumor cells are produced, replicated and co-exist with other surrounding cells including endothelial cells, immune cells, lymphocytes, adipocytes, fibroblasts, and cancer-associated fibroblasts (CAFs) (Wang et al., 2022). Recent studies indicate that immunological components in the TME promote tumor growth and invasion, regulate tumor cell immune escape, cause immunosuppression, and increase therapeutic resistance (Altorki et al., 2019; Zhao et al., 2021). For example, tumor-infiltrating immune cells (TIICs) in the TME secrete various growth factors and cytokines that promote drug resistance and suppress immune responses in different cancer types (Ammirante et al., 2010; Straussman et al., 2012; Su et al., 2018; Zhang et al., 2020). Therefore, the TME is a major cause of immunotherapy failure and its various side effects.

In PCa, the infiltration of activated dendritic cells, M2 macrophages, CD8^+^ T-cells, resting NK cells, and memory B cells is substantially correlated with the degree of malignancy (Wu et al., 2020). The proportion of tumor-infiltrating myeloid-derived immune suppressor cells (MDSCs) and B lymphocytes increases in the TME over time, and these cells, through the secretion of interleukin 23 (IL-23), drive PCa progression to castration-resistant prostate cancer (CRPC) (Ammirante et al., 2010; Calcinotto et al., 2018). The downregulation of pro-inflammatory M1 markers and upregulation of M2-associated pro-tumorigenic effectors are also associated with the progression of prostate carcinoma (Bolis et al., 2021). In particular, M2 tumor-associated macrophages contribute to the development of bone metastasis, chemotherapy resistance, and castration resistance in PCa (Kim et al., 2011). However, the specific interactive mechanism between immune-related cells and PCa has not been fully clarified. Therefore, it is important to explore the key genes in TME and construct a TIIC-related risk signature that may help predict patient outcomes in PCa and improve the understanding of the TME immunogenomic profile (Xu et al., 2021a; Xu et al., 2021b; Yang et al., 2021a). In recent years, several prognostic models based on TME-related genes have been established and have shown promising tumor prognosis abilities (Xu et al., 2021a; Xiang et al., 2021; Yang et al., 2021a), but there is a lack of comprehensive risk-scoring model based on TME/TIIC-related gene signatures for prognostic prediction of PCa recurrence.

Dendritic cells function as antigen-presenting cells in the TME, as they recognize, capture, and present tumor-associated antigens (TAA) to T-cells in secondary lymphoid organs (e.g., lymph nodes). In response to pathogen invasion, dendritic cells are activated, travel to the nearest lymph node, and present antigens to naive T-cells, consequently stimulating the proliferation of naive T-cells and inducing innate and adaptive antitumor immune responses (Chudnovskiy et al., 2019). The main types of DCs in the TME include plasmacytoid DCs (pDCs) and conventional DCs (cDCs), which are subdivided into type 1 (cDC1) and type 2 (cDC2) cDCs (Kvedaraite and Ginhoux, 2022). cDC1 can cross-present TAA bound to the major histocompatibility complex (MHC) -I to the T-cell receptor (TCR) of CD8^+^ T-cells, whereas DCs express costimulatory factors such as B7, which can bind to CD28 molecules on the surface of T-cells, providing a second signal for T-cell activation. CD8^+^ T-cells can then be induced to differentiate into cytotoxic T lymphocytes (CTL), which can specifically recognize and kill target cells accurately. cDC2 activates CD4^+^ T-cells *via* the TAA–MHC-II complex, promotes the proliferation and differentiation of CD4^+^ T-cells into helper T-cells (Th), and mediates humoral immunity (Kvedaraite and Ginhoux, 2022). These indicate that understanding dendritic cells and the interaction process in TME may help to build a prognosis model and improve the efficacy of cancer treatments in the future.

In this study, we developed a risk-score signature based on weighted gene co-expression network analysis (WGCNA), using two PCa sample groups from the PRAD datasets (GSE116918 and TCGA-PRAD). We examined the correlation between the risk signature and clinical parameters (including age, T-stage, N-stage, and Gleason score), tumor mutation burden, tumor ESTIMATE score, levels of immune checkpoint-related genes, sensitivity to immune checkpoint inhibitors (ICIs), and response to antitumor therapies. Additionally, we analyzed the enrichment of signaling pathways in different risk cohorts and investigated the potential PCa progression-related mechanisms of TIIC-related gene expression according to the identified signature.

## 2 Materials and methods

### 2.1 Collection of multi-omics data

Normalized RNA sequencing data (fragments per kilobase million, FPKM), based on mRNA samples (498 prostate cancer tissues and 52 normal tissues) from the Illumina HiSeq RNA-Seq platform of the Cancer Genome Atlas Prostatic Adenocarcinoma (TCGA-PRAD) database, were obtained from The Cancer Genome Atlas (TCGA) (https://portal.gdc.cancer.gov/) (Weinstein et al., 2013). FPKM data were transformed into transcript per million (TPM) values following log_2_ (x + 1) normalization. The corresponding clinical profiles (age, AJCC-TNM stage, and Gleason score) were downloaded from the TCGA portal, and the clinical data for progression-free survival (PFS) analysis was downloaded from TCGA Pan-Cancer Clinical Data Resource (TCGA-CDR) (Liu et al., 2018). After removing four patients without survival data, 494 patients with PRAD were selected for the present study. The clinical features of the patients are summarized in Table 1.

**TABLE 1:** Clinical co-variates of the training and validation cohorts.

Characteristics	Training Cohort (TCGA-PRAD, N = 494)			Validation Cohort (GSE116918, N = 248)		
	high risk (N = 247)	low risk (N = 247)	*p*	high risk (N = 124)	low risk (N = 124)	*p*
Age			0.037			0.894
≤65	165 (66.8%)	186 (75.3%)		44 (35.5%)	43 (34.7%)	
>65	82 (33.2%)	61 (24.7%)		80 (64.5%)	81 (65.3%)	
T-stage			<0.01			0.436
T1	0	0		25 (20.2%)	26 (21%)	
T2	66 (27.2%)	121 (49.6%)		33 (26.6%)	43 (34.7%)	
T3	171 (70.4%)	119 (48.8%)		51 (41.1%)	41 (33.1%)	
T4	6 (2.5%)	4 (1.6%)		1 (0.8%)	3 (2.4%)	
unknow	4 (1.6%)	3 (1.2%)		14 (11.3%)	11 (8.9%)	
N-stage			<0.01			
N0	161 (65.2%)	183 (74.1%)		-	-	
N1	58 (23.5%)	19 (7.7%)		-	-	
unknow	28 (11.3%)	45 (18.2%)		-	-	
M-stage						
M0	228 (92.3%)	225 (90.7%)		-	-	
M1	3 (1.2%)	0		-	-	
unknow	16 (6.5%)	23 (9.3%)		-	-	
Gleason score			<0.01			0.811
6	11 (4.5%)	34(13.8%)		20 (16.1%)	22 (17.7%)	
7	88 (35.6%)	158(64%)		48 (38.7%)	51 (41.1%)	
≥8	148 (59.9%)	55 (22.3%)		56 (45.2%)	51 (41.1%)	
Diease progress			<0.01			0.026
No progress	175 (70.9%)	226 (91.5%)		108 (87.1%)	118 (95.2%)	

The microarray data of 280 PRAD samples in the GSE116918 dataset, based on GPL25318 ([ADXPCv1a520642] Almac Diagnostics Prostate Disease Specific Array) (Affymetrix/Thermo Fisher, Belfast, United Kingdom), were downloaded from the Gene Expression Omnibus (GEO) database (http://www.ncbi.nlm.nih.gov/geo/). Detailed clinicopathological data included age, AJCC-T stage, Gleason score, metastasis-free survival (MFS) state, and MFS time. The expression data in the two platforms underwent a batch calibration process and were further normalized *via* the “sva” R package, such that they were comparable. The clinical features of the patients are summarized in Table 1. In addition, somatic mutation data of 475 patients with PRAD (based on the VarScan software) were obtained from the TCGA portal.

### 2.2 Landscape of infiltrating immune cells

The 22 TIICs constituting the TME of TCGA-PRAD samples were calculated using the CIBERSORT algorithm (http://cibersort.stanford.edu/) (Newman et al., 2015). Samples with a CIBERSORT *p*-value <0.05 were used for further study.

### 2.3 Weighted gene co-expression network analysis

The WGCNA R package was used to perform WGCNA of 19,560 gene sequences from TCGA-PRAD patients (Langfelder and Horvath, 2008). SampleTree was used to identify the outliers that were subsequently deleted. According to the mean connectivity and scale-free topology model fit, the soft threshold power (*β*) value was selected to generate a scaleless network (index of scale-free topologies = 0.90). The correlations between sample traits and candidate modules were computed to determine the models that were highly correlated with the traits. Then, similar genes were introduced into the same candidate module employing a “dynamic tree cutting” algorithm with a minimum size of 60. Correlations between the 22 TIICs and module characteristic genes were evaluated using Pearson’s correlation coefficient (*p* < 0.05). Finally, genes in the most statistically significant module were selected for subsequent analysis.

### 2.4 Construction and validation of prognostic TIIC-related gene signature

The expression levels of genes in the most statistically significant module were extracted from TCGA-PRAD and GSE116918 datasets. TCGA-PRAD was used as the training cohort, and GSE116918 was used as the validation cohort. Univariate Cox regression analysis was applied to obtain prognostic risk candidate genes from the most significant module in the “activated dendritic cells” population in the training cohort, and the genes that were significantly related to progression-free survival (PFS) (*p* < 0.01) were identified.

Then, least absolute shrinkage and selection operator (LASSO) Cox regression analysis was used to determine the best weighting coefficient of the prognostic risk candidate genes. After a 1,000-fold cross-validation of the maximum likelihood estimate of the penalty, the minimum criterion was determined using the optimal value of the penalty parameter λ. Finally, a TIIC-related gene risk signature was established, and the risk score was calculated using the following formula:

Risk score=β gene 1×expression level of gene 1+····+β gene n×expression level of gene n

Here, *ß* is the regression coefficient in the multivariate Cox regression analysis. The patients in each cohort were divided into high- and low-risk groups, based on the median risk score of the training cohort. Univariate and multivariate Cox regression analyses were performed to evaluate the independent prognostic value of the risk signature. The R package “caret” was used to randomly split TCGA-PRAD into training and test cohorts at a 7:3 ratio. Statistical significance was set at *p* < 0.05.

### 2.5 Somatic mutation analysis

The tumor mutation burden (TMB) of the TCGA-PRAD samples was visualized by the “maftools” R package (Mayakonda et al., 2018). TMB was defined as the number of base substitutions, deletions, insertions, and insertions across bases per megabase of the genome examined using non-synonymous and code-shifting indels under a 5% detection limit. Somatic alterations in PRAD driver genes were analyzed in samples with low- and high-risk scores.

### 2.6 Visualization of the expression of identified TIIC-related genes in pan-cancer from TIMER2.0

The Gene_DE module of Tumor Immune Estimation Resource version 2 (TIMER2.0; http://timer.cistrome.org) (Li et al., 2020) was used to analyze the differentially expressed TIIC-related genes between the tumor and normal tissues in pan-cancer.

### 2.7 Correlation of risk score to TME characterization

Seven methods, comprising XCELL, TIMER, QUANTISEQ, MCPcounter, EPIC, CIBERSORT, and CIBERSORT-ABS, were implemented to evaluate the extent of immune infiltration and its correlation to the risk score. The Estimation of Stromal and Immune cells in Malignant Tumors using the Expression Data (ESTIMATE) algorithm (Yoshihara et al., 2013) was used to predict tumor purity for each TCGA-PRAD sample.

### 2.8 Prediction of patient response to antitumor drug therapy

The sensitivity of PRAD samples in the high- and low-risk-score groups to antitumor drug therapy was predicted by using the R package “pRRophetic” (Geeleher et al., 2014) to estimate the half-maximal inhibitory concentration (IC50) of each sample, based on the largest publicly attainable pharmacogenomics database: the Genomics of Drug Sensitivity in Cancer (GDSC) (www.cancerrxgene.org) (Yang et al., 2013) cell line expression spectrum.

To further explore the potential role of risk score in immunotherapeutic prediction, the Cancer Immunome Database (TCIA, https://tcia.at/home) provided comprehensive immunogenomic analyses of next-generation sequencing (NGS) data for 20 solid-tumor cancers from TCGA and other data sources. The immunophenoscore (IPS) was used as a novel and robust predictor of response to anti-cytotoxic T lymphocyte antigen-4 (anti-CTLA-4) and anti-programmed cell death protein 1 (anti-PD-1) antibodies (Charoentong et al., 2017) from the downloaded TCGA-PRAD datasets. The R package “ggpubr” was used to visualize IPS in the high- and low-risk groups. Furthermore, the expression levels of 47 immune checkpoint blockade-related genes in the high-and low-risk-groups were compared, and their correlations were visualized.

### 2.9 Functional enrichment analysis

Molecular and functional relevance analyses of potential prognostic differentially expressed TIIC-related genes (PDEMRGs) were performed using Metascape (http://metascape.org) (Zhou et al., 2019). The activation of the hallmark pathway and Kyoto Encyclopedia of Genes and Genomes (KEGG) pathway, described in the MSigDB database (https://www.gsea-msigdb.org/gsea/msigdb) (Subramanian et al., 2005), was carried out to evaluate the enriched pathways in the high- and low-risk groups. To elucidate the functional annotation of each gene, including the risk signature, the gene set variation analysis (GSVA) R package was used to analyze the enrichment of the KEGG and gene ontology (GO) pathways.

### 2.10 Statistical analysis

R software (version 4.0.3) was used for all statistical analyses. The Wilcoxon test was used to compare the two groups. The R package “survivalROC” was used to calculate the area under the curve (AUC) using receiver operating characteristic (ROC) curves to identify the accuracy of the risk score. Kaplan–Meier curves with log-rank tests were used to compare survival rates. The chi-squared test was performed to correlate the risk-score subgroups with somatic mutation frequency, and Spearman analysis was used to compute the correlation coefficient. The “clusterProfiler”, “enrichplot”, “pheatmap”, and “ggplot2” R packages were used to visualize the results. Statistical significance was set at *p* < 0.05.

### 2.11 Experimental validation

Five human prostate cancer cell lines (PC-3, DU-145, C4-2, 22RV-1, and LNCAP) were used to detect mRNA levels of activated dendritic cell-related genes. Among them, the PC-3 cells and DU-145 cells were purchased from American Type Culture Collection (ATCC), 22RV-1 cells and LNCAP cells were purchased from the Shanghai Fuheng Biotechnology Co., Ltd., and C4-2 cells were a gift from the Department of Endocrinology, the first hospital of Jilin University, Changchun, Jilin, China. All cell lines were cultured in Roswell Park Memorial Institute (RPMI-1640) medium supplemented with 10% fetal bovine serum (VivaCell, Shanghai XP Biomed Ltd.,). All media were supplemented with 5000 U/mL penicillin–streptomycin (Gibco). All cell lines were grown in a humidified atmosphere containing 5% CO_2_ at 37°C. RNA was isolated using RNAiso Plus [Takara Biomedical Technology (Beijing) Co., China]. Total RNA (2.0 μg) was subjected to reverse transcription PCR (RT-PCR) using Hifair III Reverse Transcriptase (Yeasen, China) to obtain cDNA. cDNA was diluted 20-fold; then 6 μl was used for quantitative real-time polymerase chain reaction (qRT-PCR) using Hieff^®^ qPCR SYBR Green Master Mix (No Rox) (Yeasen, China). Gene expression levels were evaluated relative to GAPDH level and calculated using the 2^−ΔΔCt^ method. All samples were analyzed at least in triplicates. The primer sequences used for PCR were as follows: *STX4*, 5′- CGG​ACA​ATT​CGG​CAG​ACT​ATT -3′ (forward) and 5′- TTC​TGG​GGC​TCT​ATG​GCC​TT -3′ (reverse); *UBE2S*, 5′- CCG​ACA​CGT​ACT​GCT​GAC​C -3′ (forward) and 5′- GCC​GCA​TAC​TCC​TCG​TAG​TTC -3′ (reverse); *TMEM93*, 5′- GCC​GCC​GTC​CTG​GAT​TAT​T -3′ (forward) and 5′- GAG​GCG​AGC​AGG​TAG​AAG​AT -3′ (reverse); *EMD*, 5′- CCG​CCT​CCT​CTT​ATA​GCT​TCT -3′ (forward) and 5′- CTC​TGG​TAG​AGT​AAA​GCG​TCC​T -3′ (reverse); GCAT, 5′- CCT​CAG​CTC​TGT​CCG​CTT​TAT -3′ (forward) and 5′- GGA​TGC​CGT​CGA​TGA​TGG​AG -3′ (reverse); *NUCB1*, 5′- CAG​AAC​CAG​CAT​ACA​TTC​GAG​GC -3′ (forward) and 5′- AGT​GAC​TCC​AGA​TAA​CGC​CGT​C -3′ (reverse); and *GAPDH*, 5′- TCA​ACA​GCG​ACA​CCC​ACT​C-3′ (forward) and 5′- GCT​GTA​GCC​AAA​TTC​GTT​GTC-3′ (reverse).

## 3 Results

### 3.1 Landscape of TIICs in TCGA-PRAD

**FIGURE 1:**
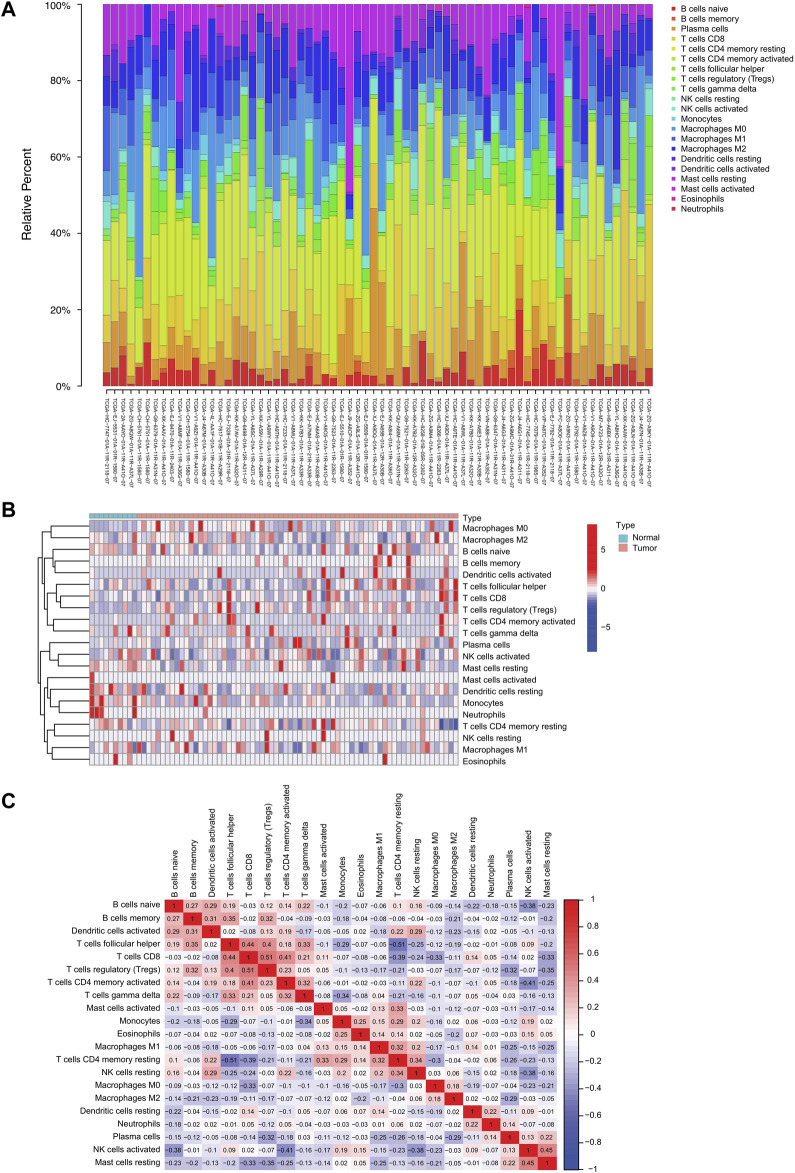
The landscape of tumor-infiltrating immune cells in prostate cancer. **(A)** Proportional heatmap of the 21 TIICs in TCGA-PRAD samples (CIBERSORT algorithm: *p* < 0.05). **(B)** Heatmap of the 21 TIICs in normal and tumor tissues from TCGA-PRAD samples. **(C)** Correlation matrix between 21 TIICs in TCGA-PRAD. Red and blue colors indicate positive and negative correlations, respectively. Color intensity corresponds to the degree of correlation.

We used the CIBERSORT algorithm to investigate 22 TIICs subsets in 550 samples (498 tumor and 52 normal) from the TCGA-PRAD dataset (Supplementary Table S1; Figure 1A). Ultimately, the abundance of 21 TIIC types from each sample was selected in each dataset (Supplementary Table S1; Figure 1A), excluding the naive T-cell type owing to its low abundance in all samples. A total of 78 samples under the threshold of the adjusted CIBERSORT *p*-value <0.05 were selected for subsequent analysis. The heatmap shows the TME patterns of 21 TIIC types in normal and tumor tissues (Figure 1B). Furthermore, the correlation matrix displays correlation coefficients between the 21 TIICs types, demonstrating a potential connection between these infiltrating immune cells in the TME (Figure 1C). Notably, CD8^+^ T-cells and regulatory T-cells (Tregs) had the strongest positive correlation (*r* = 0.51; *p* < 0.01), whereas follicular helper T-cells and resting memory CD4^+^ T-cells had the strongest negative correlation (*r* = −0.51; *p* < 0.01). In addition, the activated dendritic cells were positively correlated with several infiltrating immune cells, including memory B cells (*r* = 0.31; *p* < 0.01), naive B cells (*r* = 0.29; *p* < 0.01), and resting NK cells (*r* = 0.29; *p* < 0.01), suggesting their important roles in the TME (Supplementary Table S2).

### 3.2 Establishment of the WGCNA network

**FIGURE 2:**
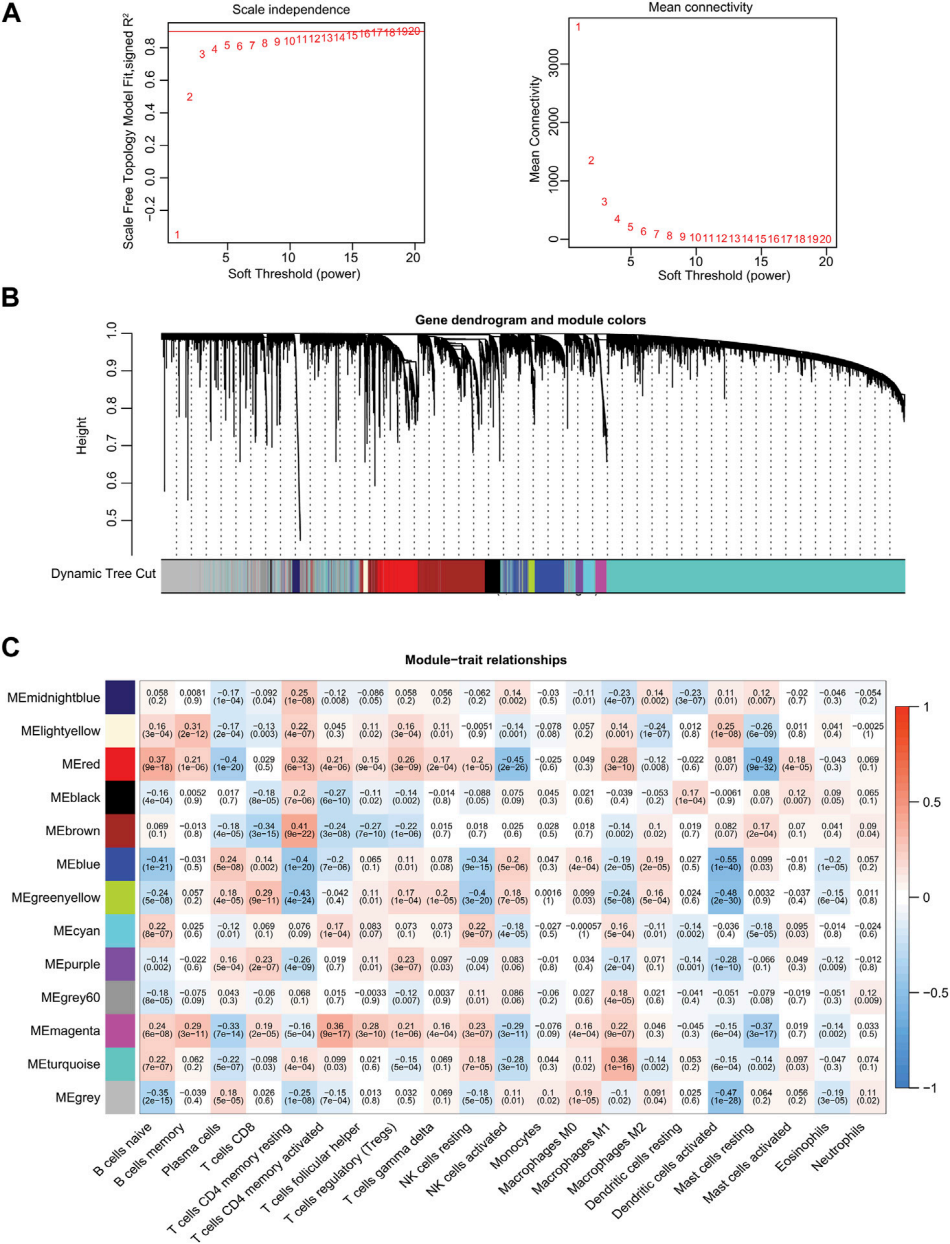
Identification of significant gene modules by WGCNA from TCGA database. **(A)** Analysis of the scale-free fit index and the mean connectivity for various soft-thresholding powers. **(B)** Hierarchical cluster dendrogram and corresponding modules using a dynamic tree-cutting algorithm. Different colors indicate different assigned modules. The gray module contains genes that cannot be assigned to any module. **(C)** Heatmap of the correlations between the assigned modules and immune-infiltrating cells (traits). Within every square, the number on the top refers to the correlation coefficient, and the number on the bottom refers to the *p*-value.

We next established a WGCNA co-expression network through the “WGCNA” R package, selecting 19,560 genes from TCGA-PRAD patient samples after gene sequencing and preprocessing. A power of *ß* = 11 was used as the best soft-thresholding parameter, with an index of scale-free topologies of *R* ^2^ = 0.90 (Figure 2A). Highly similar genes were assigned to the same module by the dynamic tree-cutting algorithm, and modules that met the hierarchical clustering analysis threshold of below 0.25 were clustered together. Consequently, 13 modules were identified in the resulting network and illustrated in a hierarchical clustering tree: MEmidnightblue, MElightyellow, MEred, MEblack, MEbrown, MEblue, MEgreenyellow, MEcyan, MEpurple, MEgrey60, MEmagenta, MEturquoise, and MEgrey (Supplementary Table S3; Figure 2B). Furthermore, the correlations between the 21 TIICs and each module were analyzed and presented as a heatmap (Figure 2C). Among these 13 modules, MEblue (*r* = −0.55, *p* = 1e^−40^), MEgrey (*r* = −0.47, *p* = 1e^−28^), and MEgreenyellow (*r* = −0.48, *p* = 2e^−30^) modules were significantly negatively correlated with activated dendritic cells (Figure 2C). Eventually, we identified the MEblue module (a total of 1,143 genes) as having the strongest correlation coefficient among the analyzed results.

### 3.3 Construction of the six-gene-based prognostic signature

**FIGURE 3:**
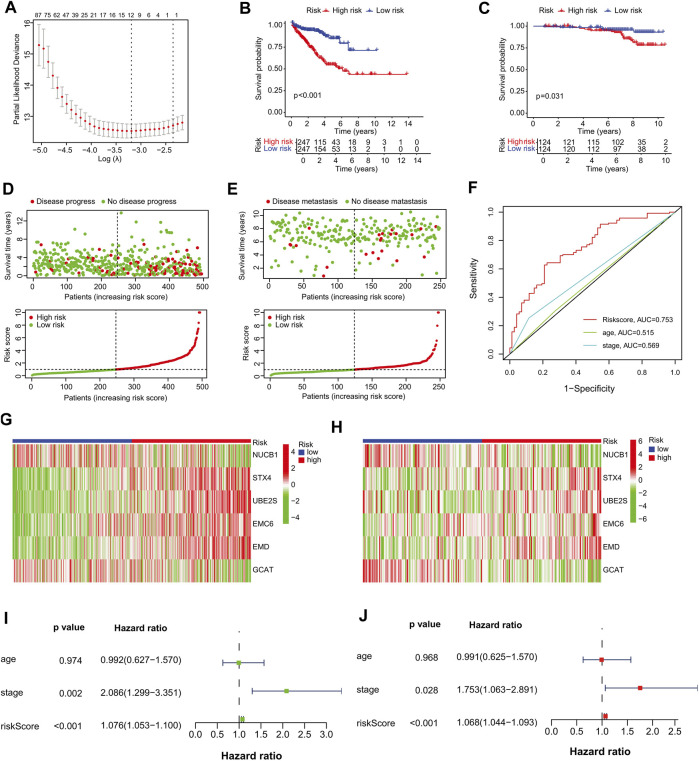
Establishment of the prognostic risk signature. **(A)** Ten-fold cross-validation for tuning parameter selection in the LASSO regression. The vertical lines are plotted based on the optimal data according to the minimum criteria and one-standard error criterion. The left vertical line represents the 12 genes identified. **(B, C)** Kaplan–Meier curve analysis comparing the PFS/MFS between patients in high-risk and low-risk groups in **(B)** training and **(C)** validation cohorts. **(D, E)** Scatter plots of the relationship between the risk-score model, patient PFS time (upper panel) and the risk curve of the risk-score growth trend (lower panel). **(D)** The training cohort, and **(E)** the validation cohort. **(F)** Survival prediction ROC curves of the risk model and other clinical indices from the training cohort. **(G, H)** Heat maps of six signature genes in the risk-score model. **(G)** The training cohort, **(H)** validation cohort. **(I–J)** Forest plots of univariate and multivariate Cox regression analyses between risk signature, other clinical data constituting the risk-score model and PFS. **(I)** Univariate Cox regression analysis, **(J)** multivariate Cox regression analysis. Squares represent hazard ratios. Bars represent 95% confidence intervals.

We selected the TCGA-PRAD dataset as the training cohort and the GSE116918 dataset as the validation cohort, the clinical features of which are listed in Table 1, and then extracted the expression levels of genes in the MEblue module from both TCGA-PRAD and GSE116918 datasets. Univariable Cox regression analysis of these genes revealed that 211 genes showed a significant prognostic value associated with PFS (*p* < 0.01) (Supplementary Table S4). LASSO Cox regression analysis of this group identified 12 genes with the highest coefficients (Figure 3A; Supplementary Table S5). Multivariate Cox regression analysis narrowed this group to six genes (*STX4*, *UBE2S*, *EMC6*, *EMD*, *NUCB1*, and *GCAT*) as the minimum set for constructing a TIIC-related genes risk signature (Table 2).

**TABLE 2:** Identification of prognostic-associated activated dendritic cell-related genes in TCGA-PRAD.

gene	coef	HR	HR.95L	HR.95H	p value
STX4	0.89140952	3.309	1.945	5.629	< 0.01
UBE2S	0.61820449	2.506	1.782	3.524	< 0.01
EMC6	0.42698353	1.993	1.411	2.815	< 0.01
EMD	0.69441052	6.405	3.095	13.254	< 0.01
NUCB1	−0.62842011	0.526	0.338	0.818	< 0.01
GCAT	−0.63160295	0.593	0.42	0.837	< 0.01

The risk formula was as follows: Risk score = (0.891 × expression level of STX4) + (0.618 × expression level of UBE2S) + (0.427 × expression level of EMC6) + (0.694 × expression level of EMD)—(0.628 × expression level of NUCB1)—(0.632 × expression level of GCAT). Among these genes, *STX4* (syntaxin 4), *UBE2S* (ubiquitin conjugating enzyme E2 S), *EMC6* (ER membrane protein complex subunit 6 or transmembrane protein 93, *TMEM93*), and *EMD* (emerin) were identified as high-risk genes, whereas *NUCB1* (nucleobindin 1) and *GCAT* (glycine C-acetyltransferase) as low-risk genes. Survival analysis showed that TCGA-PRAD patients with higher expression levels of high-risk genes had poorer prognoses than those with lower expression levels (Supplementary Figures S1A–D). The PFS of patients with higher expression levels of low-risk genes was greater than those with lower expression levels (Supplementary Figures S1E–F).

Furthermore, we analyzed the mRNA expression levels of the six TIIC-related genes from TCGA pan-cancers in the TIMER database, and found that they were significantly increased in tumor tissues compared to the adjacent normal tissues found in various cancers (*p* < 0.05) (Supplementary Figures S2, S3). For example, the high-risk genes *EMC6* and *UBE2S* were expressed to a greater extent in TCGA-PRAD tumor tissues than in normal tissues. Although *STX4* was not overexpressed in TCGA-PRAD, its mRNA level was higher in stage IV than in stages I–III (Supplementary Figure S1G). Intriguingly, we found that the mRNA levels of low-risk genes *NUCB1* and *GCAT* were elevated in tumor tissues more than those in normal tissues, but they were lower in stage IV than in stages I–III (Supplementary Figures S1H–I). Together, these results suggest that the three genes may play essential roles in the advanced disease stage and thus validate the combined influence of the six activated dendritic cell-related genes on prostate cancer risk. Presumably, *EMC6* and *UBE2S* are overexpressed in tumor tissues compared with normal tissues. They are thus likely to be oncogenic from the start, whereas *STX4* probably do not play a role during the initial stages of cancer, and *NUCB1* and *GCAT* are the significant players during this phase.

### 3.4 Validation of the prognostic performance of the six-gene risk signature in PCa

To further assess outcome prediction, we calculated the risk scores for each patient in the training cohort using the six-gene formula model and divided them into high- and low-risk groups based on the median risk score cutoff value of 0.9773 (*p* < 0.001). Kaplan–Meier analysis showed that PFS was shorter in the high-risk group than in the low-risk group in both the training (*p* < 0.001) and validation (*p* = 0.031) cohorts (Figures 3B, C). Scatter plots and risk curves indicated that the signature gene risk score was negatively proportional to the PFS of PCa patients (Figures 3D, E). These results suggest that the risk signature of the six TIIC-related genes has an excellent prediction performance and thus demonstrates the potential as a prognostic factor in PCa patients.

Next, a 5-year ROC curve analysis was performed, revealing that the ROC of the risk score (5-year PFS AUC, 0.753) was significantly higher than that of the prognostic-related clinical parameters, such as stage (0.569) (Figure 3F), validating the accuracy of the risk model. Furthermore, heatmap analysis showed that, in the high-risk group, the mRNA expression levels of *STX4*, *UBE2S*, *EMC6*, and *EMD* were higher, whereas those of *NUCB1* and *GCAT* were lower than those in the low-risk group (Figures 3G, H), suggesting their prognostic-specific roles in PCa patients. In addition, univariate and multivariate regression analyses indicated that the risk signature was an independent prognostic predictor for PFS, with hazard ratios of 1.076 (95% CI:1.053–1.100) in the univariate analysis and 1.068 (95% CI:1.044–1.093) in the multivariate analysis (Figures 3I, J). To further validate the stability of the model, we randomly split TCGA-PRAD into training and test cohorts at a 7:3 ratio and constructed another prognostic model with different variables. We found that the new model had four genes similar to our previous risk signature and accurately predicted the prognosis in the TCGA test and GSE116918 cohorts (Supplementary Table S6; Supplementary Figures S4A–F).

**FIGURE 4:**
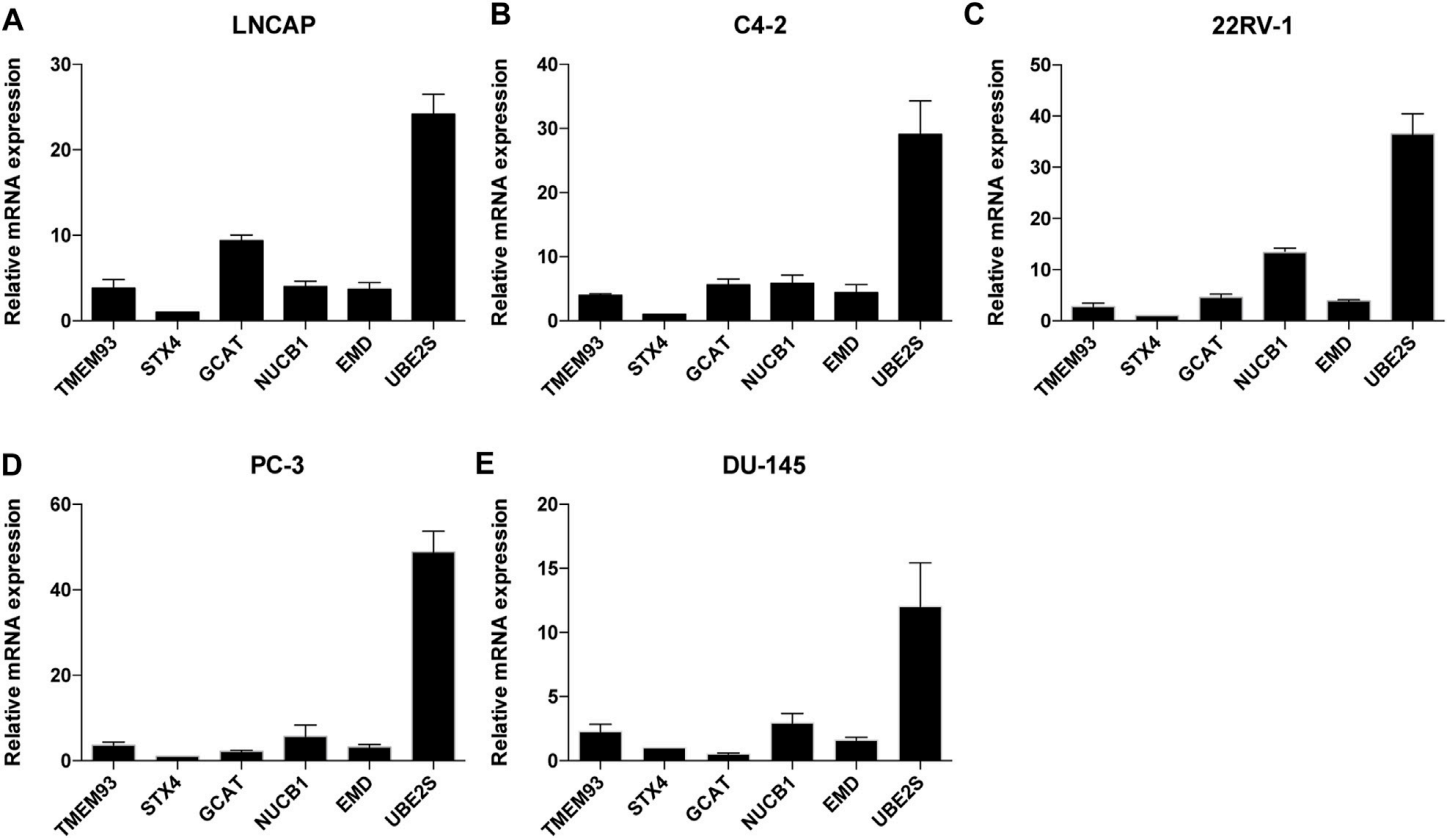
The relative mRNA expression levels of the six TIIC-related genes in five PCa cell lines. The mRNA expression level of TMEM93, STX4, GCAT, NUCB1, EMD and UBE2S in LNCAP(A), C4-2(B), 22RV-1(C), PC-3(D), DU145(E) cells. Data represent mean ± SEM (n = 3 independent experiments).

In addition, we examined the mRNA expression levels of the six signature genes in five different PCa cell lines and found that *UBE2S* had the highest expression level among the six genes (Figure 4).

### 3.5 Correlation between risk signature and PRAD clinicopathological variables

**FIGURE 5:**
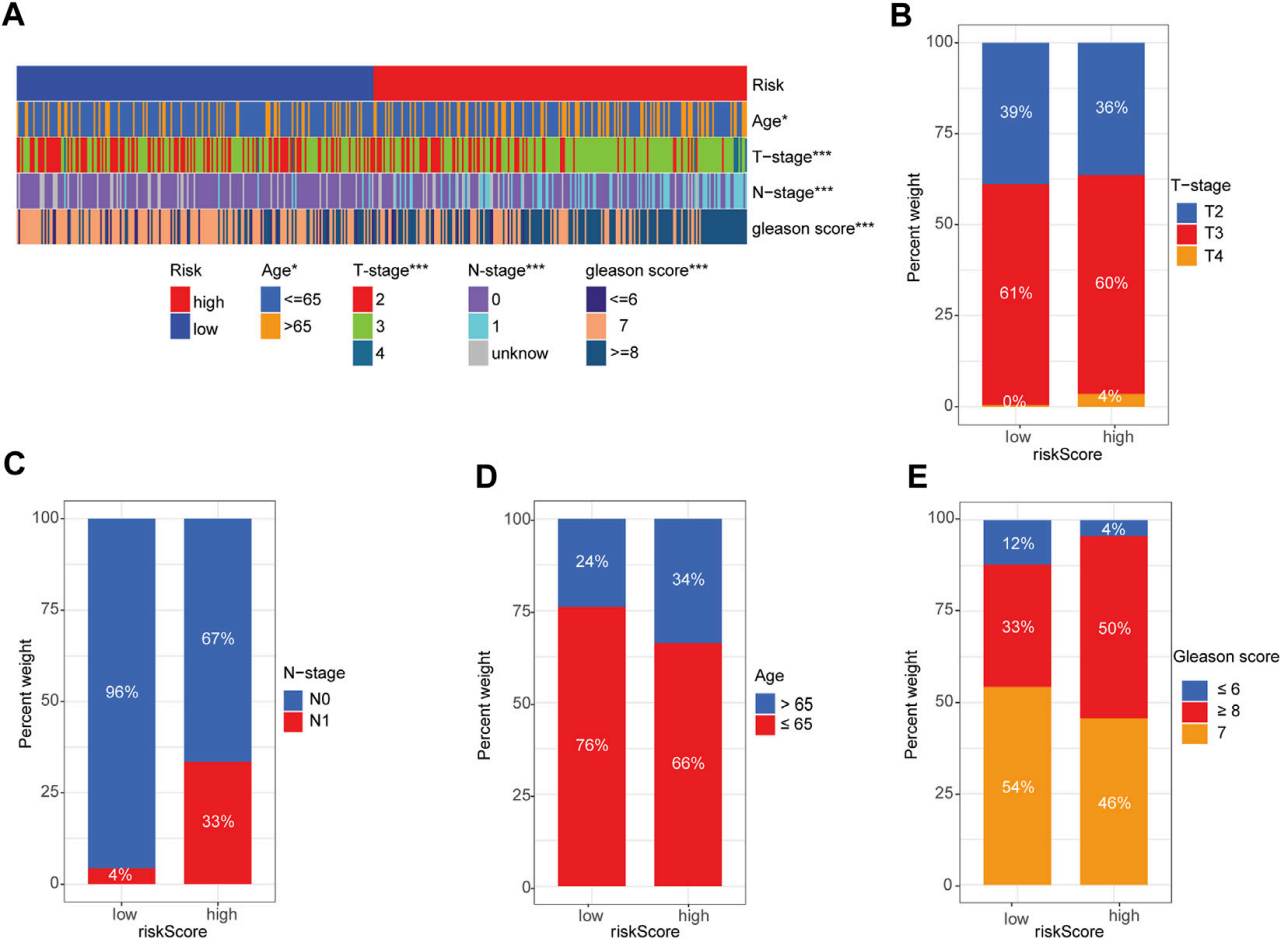
Correlation between signature gene risk score and PRAD clinicopathology variables. **(A)** Heatmap of the distribution of clinical features and corresponding risk score in each sample. **(B–E)** Rate of clinical variables subtypes in high- and low-risk-score groups: **(B)** T-stage, **(C)** N-stage, **(D)** Age, **(E)** Gleason score.

Next, we used a heatmap to visualize the distribution difference of clinical variables between the high- and low-risk groups and found that most patients in the high-risk group were older (>65 years), T-Stage scores (≥3), and N1-stage and Gleason scores (≥7) than those in the low-risk group (Figure 5A). Bar plots also confirmed the proportion difference in clinical subtypes based on age and T-stage, N-stage, and Gleason scores in the high- and low-risk groups (Figures 5B–E). Furthermore, we performed subgroup analysis to determine whether our risk signature could identify different prognoses. When patients were classified based on age, our risk signature accurately predicted patient outcomes, with higher scores indicating poorer outcomes (Supplementary Figures S5A, B). The risk signature was consistently capable of prognostically predicting patients in the T3 category (Supplementary Figure S5D), those with N0 status (Supplementary Figure S5F), and those with a Gleason score of 7 (Supplementary Figure S5I). Notably, the insignificant prognostic prediction of the risk signature in some of the clinicopathological parameter subgroups (i.e., Gleason score of 6, T-stage of T4, and N-stage of N1) was likely due to the relatively low number of cases in the study. However, it is worth noting that our risk signature demonstrated better predictions for PRAD patients in the late stages of PCa than for those in the early stages (i.e., Gleason score >6 and > T2 stage). These findings, combined with the results of the univariate and multivariate regression analyses (Figures 3I, J), indicate the statistical and clinical significance of the risk signature as a prognostic, predictive indicator.

### 3.6 Correlation between risk score and TMB in TCGA-PRAD

**FIGURE 6:**
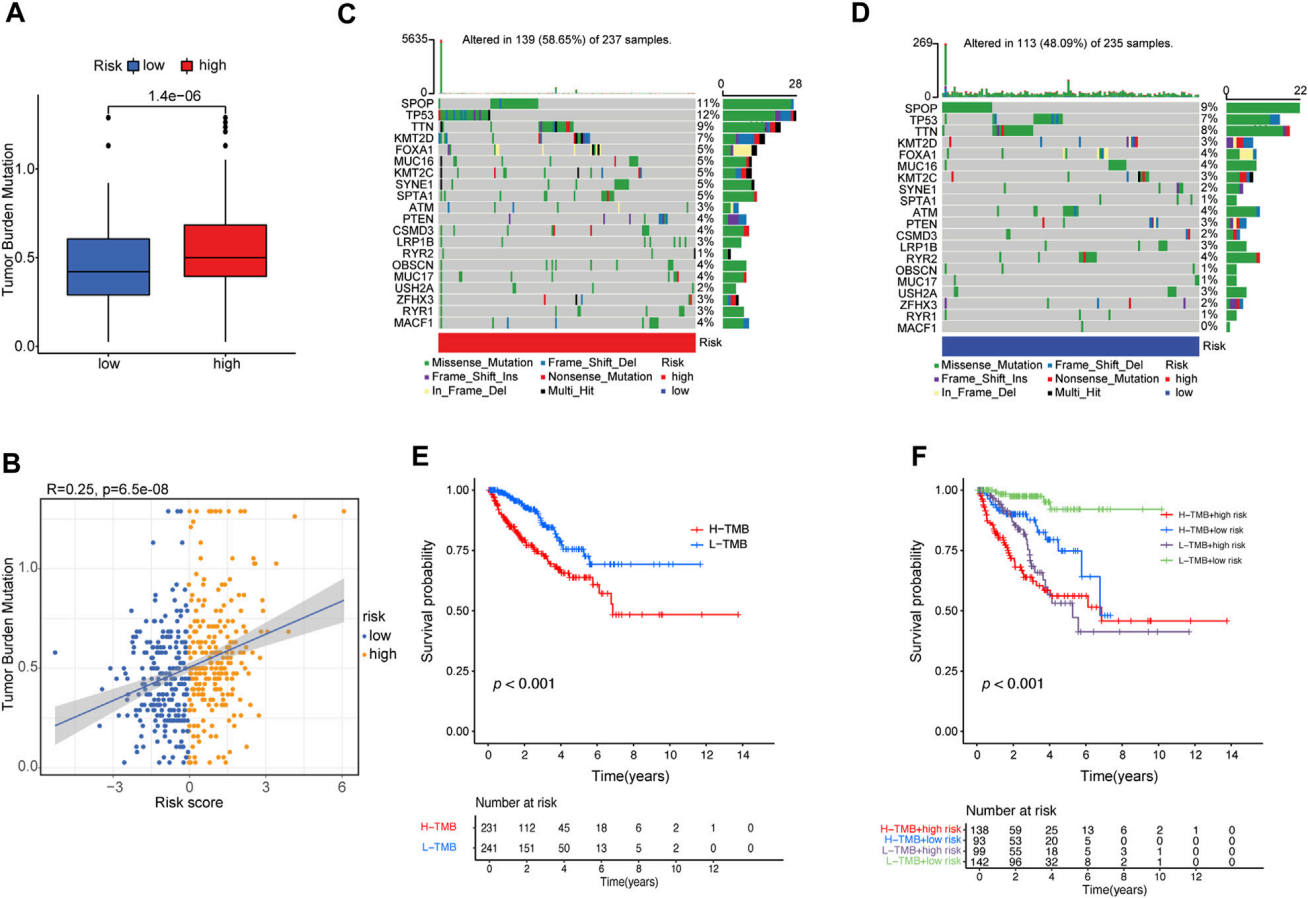
Correlation between risk score and TMB. (A) The difference in TMB between patients from the high- and low-risk score subgroups. **(B)** Scatterplots depicting the positive correlation between risk scores and TMB. The oncoPrint was constructed using the **(C)** high-risk score and **(D)** low-risk score subgroups. **(E)** Kaplan–Meier curves for high- and low-TMB subgroups. **(F)** Kaplan–Meier curves for patients stratified by both TMB and risk score.

PCa frequently exhibits genomic alterations (to the AR axis, ETS family, TP53, PTEN, or RB) (Cancer Genome Atlas Research, 2015; Abida et al., 2019). These include alterations in genes involved in biochemical pathways (the PI3K/AKT/MAPK pathway and cell cycle-related pathways) (Cancer Genome Atlas Research, 2015; Abida et al., 2019; Powles et al., 2022), epigenetic changes (Cancer Genome Atlas Research, 2015; Abida et al., 2019), alterations in DNA repair pathways, including homologous recombination repair (HRR) and mismatch repair (MMR) (Cancer Genome Atlas Research, 2015; Abida et al., 2019; Devlies et al., 2020), and single-nucleotide variants (SNVs) (AR, TP53, PI3KCA, BRCA2, PTEN, APC, CDK12, and ATM) (Robinson et al., 2015; Abida et al., 2019; Powles et al., 2022). TMB is calculated based on the somatic mutation frequency and has been proposed as a predictor of immunotherapy efficacy in various cancers, including bladder cancer, NSCLC, small cell lung cancer, and melanoma (Rizvi et al., 2015; Yarchoan et al., 2017). We compared the TMB of patients in the low- and high-risk groups and found that the TMB of the high-risk group was significantly higher than that of the low-risk group (*p* = 1.4e^−6^) (Figure 6A). The risk score positively correlated with TMB (R = 0.25, *p* = 6.5e^−8^; Figure 6B).

Furthermore, we examined somatic variations in driver genes in PCa between the low-risk and high-risk groups, and the top 20 genes with the highest mutation frequencies were analyzed (Figures 6C, D). We found that the *SPOP* (speckle-type POZ protein) gene was the most significantly mutated gene (SMG) in both risk-score subgroups (11% and 9%) (Figures 6C, D), confirming previous reports that *SPOP* is the most frequently mutated gene in PCa (Jin et al., 2021). *TP53* (12% vs. 7%) had higher somatic mutation rates in the high-risk group, whereas *RYR2* (4% vs. 1%) had higher somatic mutation rates in the low-risk group. These findings demonstrate the genetic profiles underlying the intrinsic connection between activated dendritic cell infiltration and somatic variants in PCa immunotherapy.

Then, the patients were separated into a high-TMB group (*n* = 231) and a low-TMB group (*n* = 241) based on the optimal cutoff value of TMB (cutoff value = 0.474). Kaplan–Meier curves showed that patients in the low-TMB group had better PFS than those in the high-TMB group (log-rank test, *p* ≤ 0.001; Figure 6E). To evaluate the synergistic effect of the TMB grouping and risk-score grouping in the prognostic stratification, patients were divided into subgroups of high-TMB and high-risk, high-TMB and low-risk, low-TMB and high-risk, and low-TMB and low-risk groups based on the optimal cutoff value of TMB and median risk score cutoff value. The TMB status did not affect the survival prognosis prediction based on the risk-score group. However, the risk-score subgroup showed significant survival differences in both the low- and high-TMB groups (log-rank test, high-TMB and high-risk vs. high-TMB and low-risk, *p* < 0.001; low-TMB and high-risk vs. low-TMB and low-risk, *p* < 0.001; Figure 6F). Notably, the low-TMB and low-risk subgroups had the best PFS rates, whereas the low-TMB and high-risk subgroups had the worst PFS rates (Figure 6F).

### 3.7 Correlation between risk signature and TME context of PRAD

**FIGURE 7:**
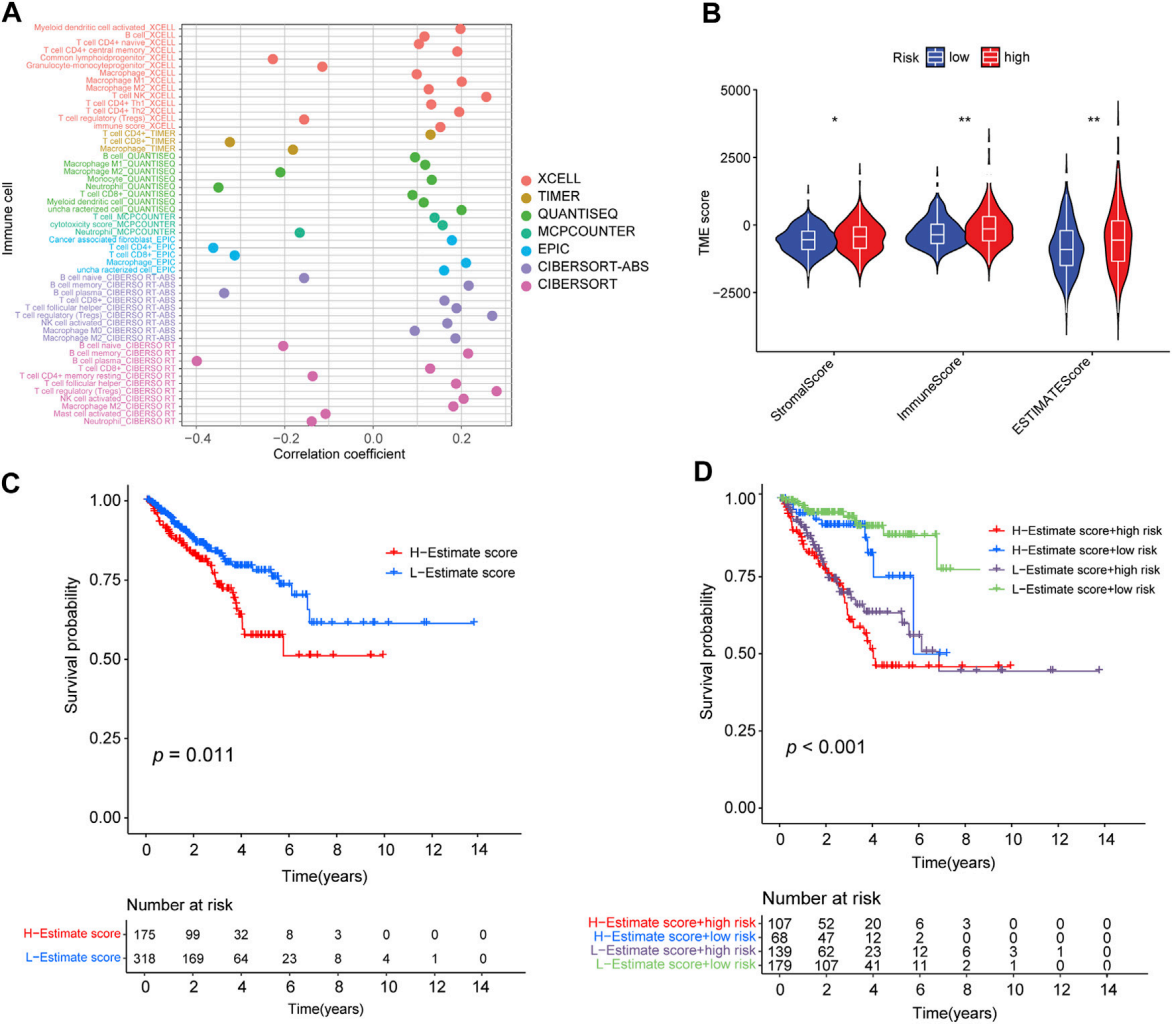
Correlation between the abundance of tumor-infiltrating cells and risk score. **(A)** Spearman correlation analysis shows that patients in the high-risk group were more positively associated with tumor-infiltrating immune cells. **(B)** Distribution of stromal score, immune score, and ESTIMATE score in high- and low-risk groups (*: *p*-value ≤ 0.05; **: *p*-value ≤ 0.01). **(C)** Kaplan–Meier curves for high- and low-ESTIMATE score subgroups. **(D)** Kaplan–Meier curves for patients stratified by both ESTIMATE score and risk score.

To investigate the intrinsic and intimate connection between the risk signature and TIICs in the TME, we performed a correlation analysis of the risk score in the TME context of PRAD and found that the risk score had a strong, negative correlation with subpopulations of naive B cells (CIBERSORT, *R* = −0.2, *p* < 0.01), plasma B cells (CIBERSORT, *R* = −0.4, *p* < 0.01), and neutrophil cells (QUANTISEQ, *R* = −0.35, *p* < 0.01) but was positively correlated with an abundance of B cells (XCELL, *R* = 0.12, *p* < 0.01), memory B cells (CIBERSORT-ABS, *R* = 0.22, *p* < 0.01), M1 Macrophages (XCELL, *R* = 0.2, *p* < 0.01), activated NK cells (CIBERSORT-ABS, *R* = 0.17, *p* < 0.01), activated myeloid dendritic cells (CIBERSORT-ABS, *R* = 0.19, *p* < 0.01), activated myeloid dendritic cells (XCELL, R = 0.2, *p* < 0.01), and follicular helper T-cells (CIBERSORT, R = 0.19, *p* < 0.01) (Supplementary Table S7; Supplementary Figures S5A, B). We also analyzed the correlation between the risk signature and immune infiltration (Figure 7A). ESTIMATE analysis showed that the immune (*p* < 0.01), stromal score (*p* < 0.05), and ESTIMATE scores (*p* < 0.01) were significantly higher in the high-risk group than in the low-risk group (*p* < 0.01) (Figure 7B).

In addition, we determined the optimal cut-off value of the ESTIMATE score (−390.9762) using the minimum *p*-value method and classified the patients into a high-ESTIMATE group (*n* = 175) and low-ESTIMATE group (*n* = 318). Kaplan–Meier curves showed that patients in the low-ESTIMATE group had better PFS rates than those in the high-ESTIMATE score group (*p* = 0.011; Figure 7C). Furthermore, we analyzed the synergistic effect of the ESTIMATE score and risk-score grouping in the prognostic stratification. Kaplan–Meier curves indicated that the ESTIMATE score did not affect the survival prognosis prediction based on the risk-score subgroup. The risk-score subgroup demonstrated significant survival differences in low and high ESTIMATE subgroups (log-rank test, high-ESTIMATE and high-risk vs. high-ESTIMATE and low-risk, *p* < 0.001; low-ESTIMATE and high-risk vs. low-ESTIMATE and low-risk, *p* < 0.001; Figure 7D). Notably, the low-ESTIMATE and low-risk subgroups had the best PFS rates, and the high-ESTIMATE and high-risk subgroups had the worst PFS rates (Figure 7D).

### 3.8 Correlation between risk score and efficacy of antitumor therapeutic drugs

**FIGURE 8:**
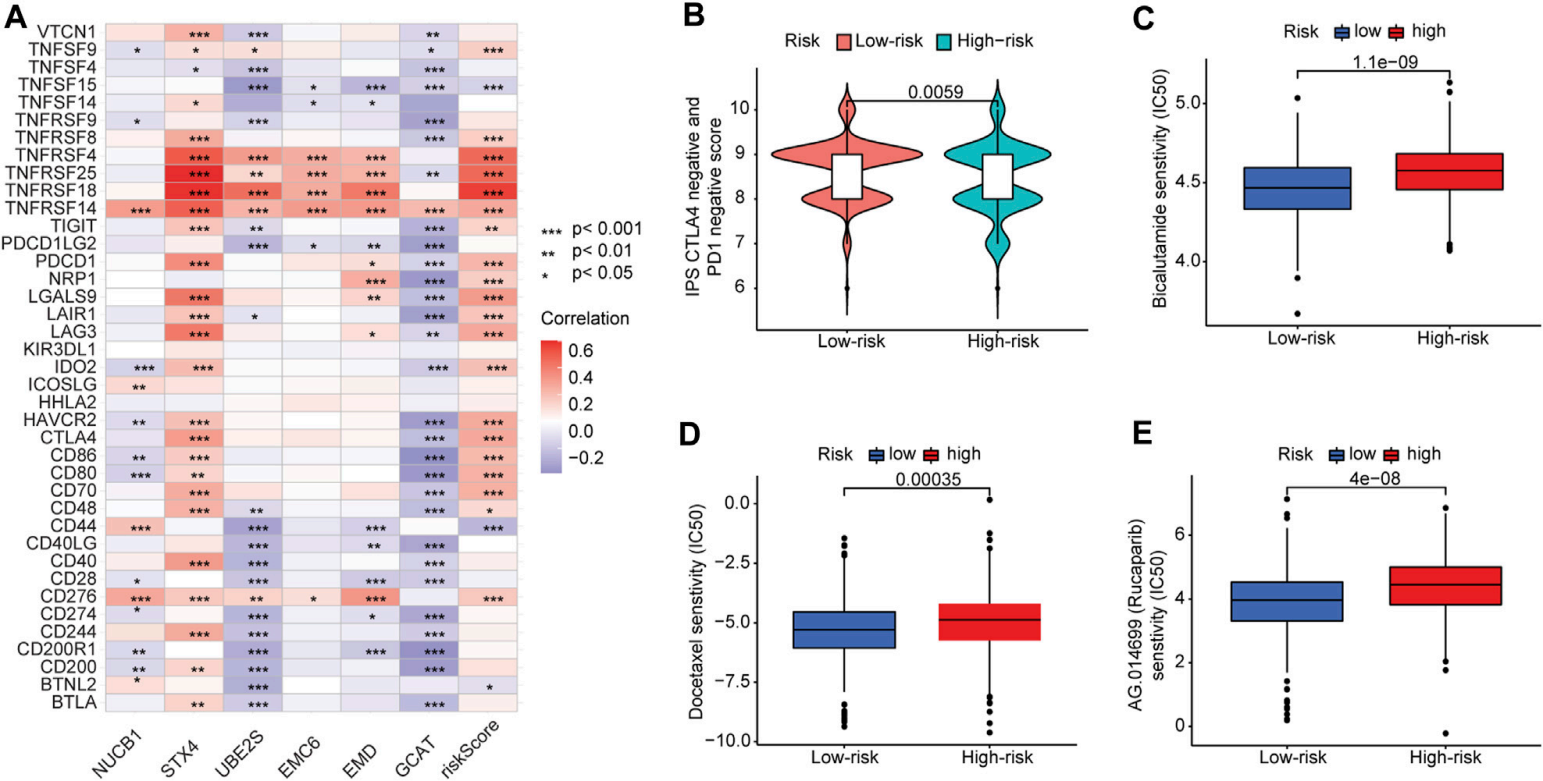
Correlation between risk score and immune checkpoint blockade genes and efficacy of antitumor drug therapy in PCa. **(A)** Correlation of expression levels of immune checkpoint blockade genes with the risk score. **(B)** Violin plot of IPS scores distribution in two groups. **(C)** Sensitivity analysis of bicalutamide in patients in high- and low-risk-score groups. **(D)** Sensitivity analysis of docetaxel in patients in high- and low-risk-score groups. **(E)** Sensitivity analysis of rucaparib in patients in high- and low-risk-score groups.

We further analyzed the association between the risk score and efficacy of antitumor therapeutic drugs for PCa. Although many immune checkpoint blockade-related genes (e.g., *PDCD1* and *CTLA4*) showed a significantly positive correlation with the risk score (Figure 8A), the scores of the IPS-PD1 blocker, IPS-CTLA4 blocker did not reveal significant differences between the high-risk and low-risk groups (Supplementary Figures S7A–C). Intriguingly, the IPS score (PD-1 negative and CTLA-4 negative; Figure 8B) of the high-risk group was higher than that of the low-risk group, suggesting that patients in the high-risk group could have benefited from immune checkpoint blockade (ICB) treatment instead of PD1/CTLA4 immunotherapy. Furthermore, we used the pRRophetic package to evaluate the sensitivity of the risk score to antitumor therapeutic drugs. We found that three common PCa drugs (bicalutamide, docetaxel, and rucaparib/AG.014699) showed different sensitivities in the high-risk and low-risk groups. Furthermore, patients in the high-risk group had higher half-inhibitory concentration (IC50) values than those in the low-risk group, indicating that they were less sensitive to these three antitumor therapeutic drugs (Figures 8C–E).

### 3.9 Enrichment of signaling pathways in high- and low-risk groups

**FIGURE 9:**
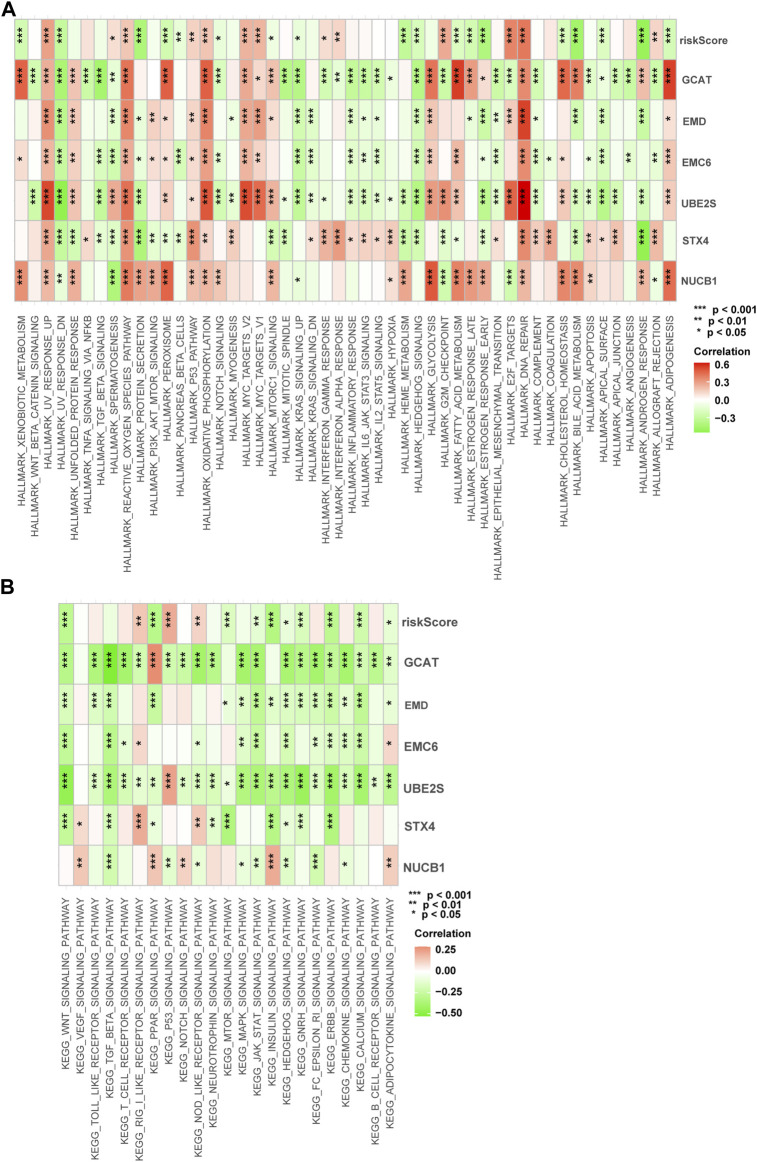
Enrichment pathways of GSVA. **(A)** Correlation of risk score with the representative pathway terms of Hallmark. **(B)** Correlation of risk score with the representative pathway terms of KEGG.

We performed gene set variation analysis (GSVA) in the training cohort to analyze the signal pathways activated in high-risk or low-risk groups (Figures 9A, B). In the high-risk group, E2F-regulated, DNA repair, MYC-regulated, UV-activated, glycolysis, and p53-mediated signaling pathways were activated. These signaling pathways are involved in advanced disease progression (Mandigo et al., 2022), sensitivity to drug therapies (Wei et al., 2021), drug resistance (Jividen et al., 2018), tumor immune infiltration (Elliott et al., 2019), tumor proliferation (Elliott et al., 2019), and immune resistance (Cascone et al., 2018). In the low-risk subgroup, the WNT, PPAR, protein secretion, cholesterol homeostasis, and androgen response signaling pathways were elevated. These signaling pathways are involved in the regulation of the disease metastasis (Leibold et al., 2020), the neuroendocrine differentiation (Liu et al., 2019), and the cell cycle, proliferation, and migration (Aurilio et al., 2020). These results show differences in the biological processes between the high-risk and low-risk groups.

The endomembrane system organization process was the most significantly enriched gene ontology biological process (GO-BP) among the six TIIC-related genes (Supplementary Figure S5A). We also used gene set enrichment analysis (GSEA) to analyze the functional enrichment activated by the six TIIC-related genes and identified significant enrichments in both the GO pathways (Supplementary Table S8; Supplementary Figures S5B–G) and KEGG pathways (Supplementary Table S9; Supplementary Figures S6A–F).

## 4 Discussion

In this study, to address the research gap regarding effective prognostic indicators in PCa, we constructed a TIIC-related gene risk signature to predict the PFS of PCa patients based on PCa datasets from TCGA-PRAD and GSE116918. Our risk model was sensitive, specific, and reliable for predicting PFS in PCa, indicating its potential for clinical use and hence warrants further investigation.

In recent years, dendritic cells have been explored as promising candidates for vaccination protocols for cancer treatment (Ahmed and Bae, 2014). The FDA approved the cancer vaccine Sipuleucel-T (Provenge) for treating asymptomatic metastatic castrate-resistant prostate cancer (mCRPC) in 2010. Sipuleucel-T is a vaccine made from patient-isolated dendritic cells with known prostate tumor-associated antigens and targets explicitly prostatic acid phosphatase (PAP) (Li et al., 2021). PROSTVAC, an active immunotherapy vaccine, can induce the immune response of tumor-infiltrating T-cells by targeting prostate-specific antigen (PSA) and demonstrates high potency and low adverse effects against PCa patients with low disease burden and indolent disease (Gulley et al., 2019). However, treatment has a limited impact on the median overall survival or survival without events in patients with mCRPC (Gulley et al., 2019). Therefore, properly selecting targeted antigens and adjuvant components can be critical for overcoming immune resistance within the TME. MDSCs, and M2-tumor-associated macrophages have been found to drive tumor progression in PCa (Ammirante et al., 2010; Bolis et al., 2021). Still, the exact role of dendritic cells in the development and progression of PCa remains largely unknown. In this study, we established a prognostic risk signature based on a module most significantly correlated with activated dendritic cells. We found that the risk signature was an independent indicator of PCa recurrence, indicating that activated dendritic cells are critical to helping generate antitumor immunity in the TME.

We identified six genes in the risk signature—*STX4*, *UBE2S*, *EMC6*, *EMD*, *NUCB1*, and *GCAT*—as the most critical TIIC-related prognostic genes in PCa patient samples. The protein encoded by *STX4* is a membrane protein essential for activating dendritic cells (Verboogen et al., 2017), activating human plasma cells to secrete antibodies (Gómez-Jaramillo et al., 2014), and promoting breast tumor cells invasion and metastasis (Brasher et al., 2022). The elevated expression level of *STX4* is also correlated with poor prognosis in the clear cell renal carcinoma (He et al., 2021). *UBE2S* encodes a ubiquitin-conjugating enzyme involved in protein degradation and signal transduction. The UBE2S protein plays an oncogenic role in various tumors, including urinary bladder cancer (Tang et al., 2021), breast cancer (Ayesha et al., 2016), endometrial cancer (Lin et al., 2019), ovarian cancer (Hu et al., 2021), lung cancer (Liu and Xu, 2018; Qin et al., 2020), colorectal cancer (Li et al., 2018), hepatocellular carcinoma (Gui et al., 2021), and melanoma (Wang et al., 2021), *via* the activation of the mTOR pathway (Tang et al., 2021), SOX6/β-Catenin signaling pathway (Lin et al., 2019), and Wnt/β-catenin signaling pathway (Qin et al., 2020; Hu et al., 2021). Previous studies have found that EMC6 protein levels are reduced in gastric cancers (Wang et al., 2017; Li et al., 2019) but are significantly elevated in cervical cancers (Shen and Ding, 2017), suggesting that the protein may act as either a tumor suppressor or promoter, depending on the cancer type (Shen and Ding, 2017). The expression level of *EMD* is elevated (compared with that in normal tissues) in invasive breast carcinoma, head and neck squamous cell carcinoma, esophageal carcinoma, cholangiocarcinoma, hepatocellular liver carcinoma, lung adenocarcinoma/squamous carcinoma, and rectal adenocarcinoma, according to analysis in the TIMER database. However, lower expression level of *EMD* is associated with tumor aggressiveness in the osteosarcoma (Urciuoli et al., 2020). Previous reports have shown that the downregulation of *NUCB1* in pancreatic ductal adenocarcinoma indicates poor prognosis (Hua et al., 2021), and the N-terminal DNA-binding domain of NUCB1 can bind to canonical E-box sequences and induce cell epithelial–mesenchymal transition (Sinha et al., 2019). Some genes are highly expressed in tumors and positively correlate with prolonged prognosis (Hu et al., 2018; Cao et al., 2021). In our risk signature, we found that the low-risk genes *NUCB1* and *GCAT* were higher in tumor tissues than in adjacent normal tissues in PRAD; however, their expression levels in the high stage were lower than those in the low stage and that the risk signature performed well in prognosis prediction. *GCAT* is ubiquitously expressed in the pancreas and prostate and is overexpressed in uterine corpus endometrial carcinoma, PRAD, lung adenocarcinoma/squamous carcinoma, invasive breast carcinoma, and colon adenocarcinoma. GCAT is primarily involved in amino acid metabolism as a low-risk gene and is overexpressed in PRAD; however, the specific mechanism of *GCAT* requires further study. We examined the mRNA expression levels of the six signature genes in PCa cell lines and found that *UBE2S* had the highest expression level among the six genes.

We found that a combination model of six TIIC-related genes could be used as a risk signature for predicting prognosis and PCa risk. This risk signature revealed that the high- and low-risk groups had differentially enriched pathways with distinct molecular mechanisms for tumorigenicity and progression, indicating the oncogenic functions of the six TIIC-related genes in PCa.

The six TIIC-related gene signatures we identified were used to separate the PCa patients into high- and low-risk groups, with significant differences in clinicopathology and prognosis. PCa patients in the high-risk group had higher IPS scores (PD-1 negative and CTLA-4 negative), significantly correlated with immune checkpoint blockade-related genes (i.e., *TNFRSF4*, *TNFRSF14*, *TNFRSF18*, and *TNFRSF25*). In contrast, the low-risk group was strongly associated with *CD44* and *CD200R1* genes. This finding indicates that patients in different risk groups may benefit from targeted immune checkpoint therapies. In addition, we analyzed the correlation between our risk signature and the efficacy of several drug treatments. We found that patients in the high-risk group may benefit from immune checkpoint blockade (ICB) treatment more than PD1/CTLA4 immunotherapy, but they may respond less sensitively to docetaxel, bicalutamide, and rucaparib therapy than those in the low-risk group. Furthermore, TMB has been associated with cancer immunotherapeutic response and cancer prognosis (Burr et al., 2017; Osipov et al., 2020) because high TMB may lead to greater production of neoantigens and subsequent activation of the immune response to ICIs (Coulie et al., 2014; Rizvi et al., 2015). We found a positive correlation between the TIIC-related gene risk signature and the TMB subgroups of PCa patients, suggesting that specific immunotherapies may be more effective for PCa patients in different TMB groups.

Our study may have several limitations. First, this risk model is based on PCa patient data from TCGA and GEO datasets, mainly collected from developed countries. Thus, the risk-score model requires further validation in multicenter clinical trials and prospective studies from different regions. Second, additional experiments are needed to study the biological functions and mechanistic roles of the six TIIC-related genes in PCa.

## Data Availability

The original contributions presented in the study are included in the article/Supplementary Material, further inquiries can be directed to the corresponding author.

## Abbreviations

ADT, androgen deprivation therapy; anti-CTLA-4, anti-cytotoxic T lymphocyte antigen-4; anti-PD-1, anti-programmed cell death protein 1; CAFs, cancer-associated fibroblasts; CRPC, castration-resistant prostate cancer; FPKM, fragments per kilobase million; GEO, Gene Expression Omnibus; ICIs, immune checkpoint inhibitors; LASSO, Least Absolute Shrinkage and Selection Operator; MDSCs, myeloid-derived immune suppressor cells; MFS, metastasis-free survival; PCa, prostate cancer; PFS, progression-free survival; PRAD, prostate adenocarcinoma; TAA, tumor-associated antigens; TCGA, The Cancer Genome Atlas; TIICs, tumor-infiltrating immune cells; TMB, tumor mutation burden; TME, tumor microenvironment; WGCNA, weighted gene co-expression network analysis.
