# Supplementary figures and images for: Development and validation of a tumor immune cell infiltration-related gene signature for recurrence prediction by weighted gene co-expression network analysis in prostate cancer
Source: Front Genet. 2023 Mar 16;14:1067172. doi: 10.3389/fgene.2023.1067172 (PMC10061146; doi:10.3389/fgene.2023.1067172)

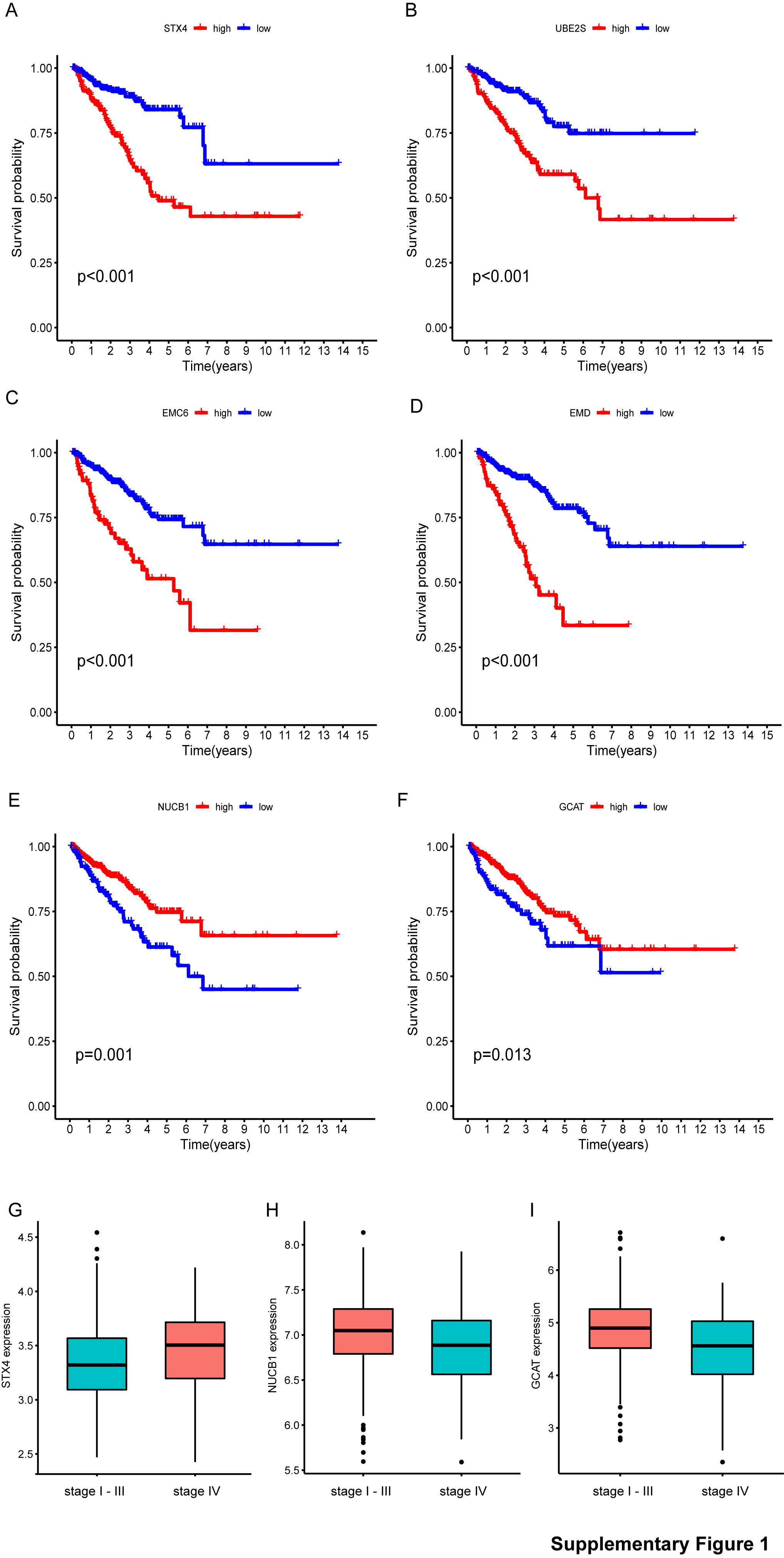

Supplement: Supplementary file 1 [file DataSheet1.ZIP › SI/Supplementary Figure 1. KaplanΓÇôMeier survival analysis of six TIIC-related genes in PRAD and the expression level of three genes in different stages.tif]

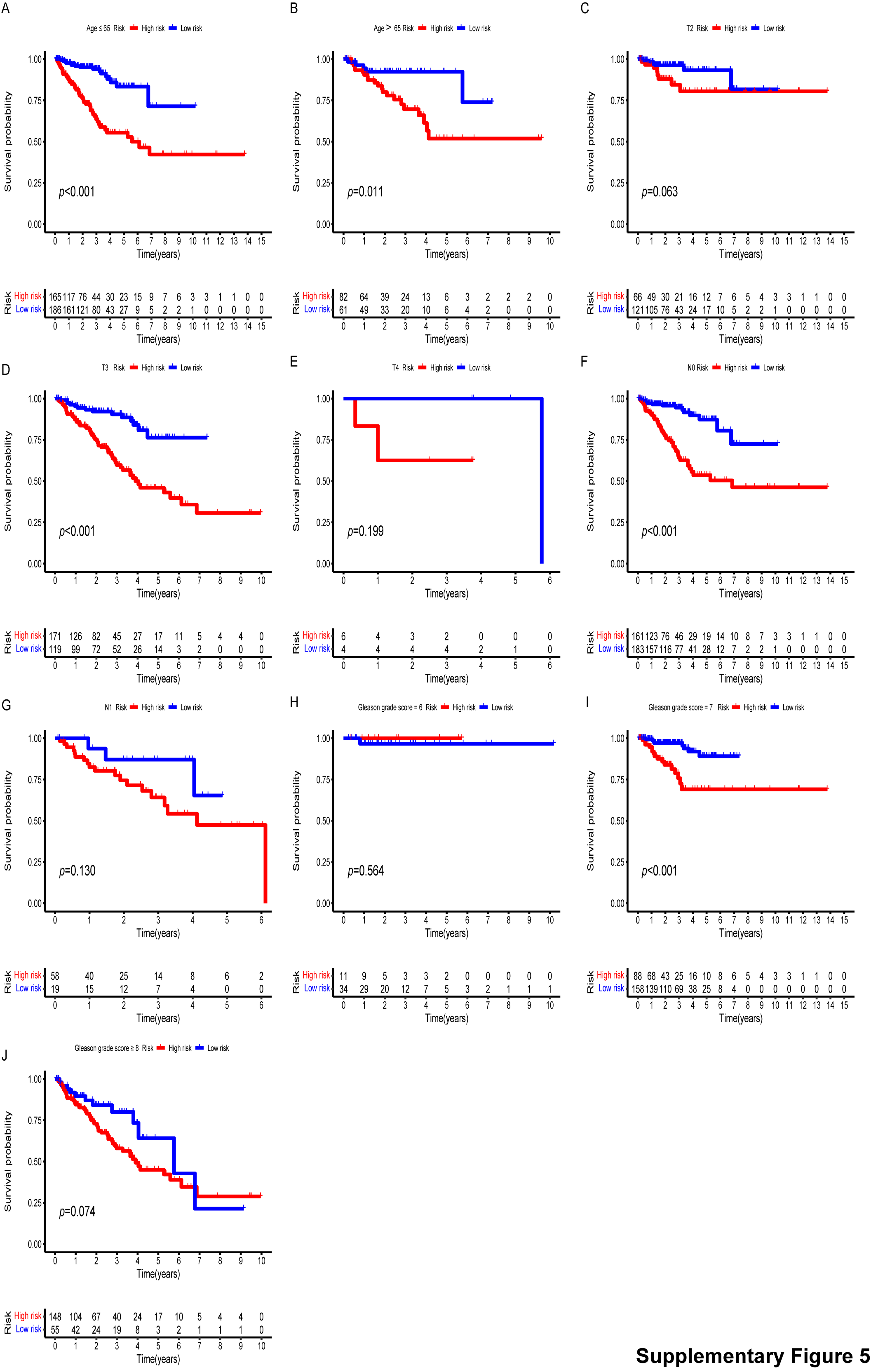

Supplement: Supplementary file 1 [file DataSheet1.ZIP › SI/Supplementary Figure 5. KaplanΓÇôMeier survival analysis for multiple PRAD subgroups according to the risk signature stratified by clinical variables.tif]

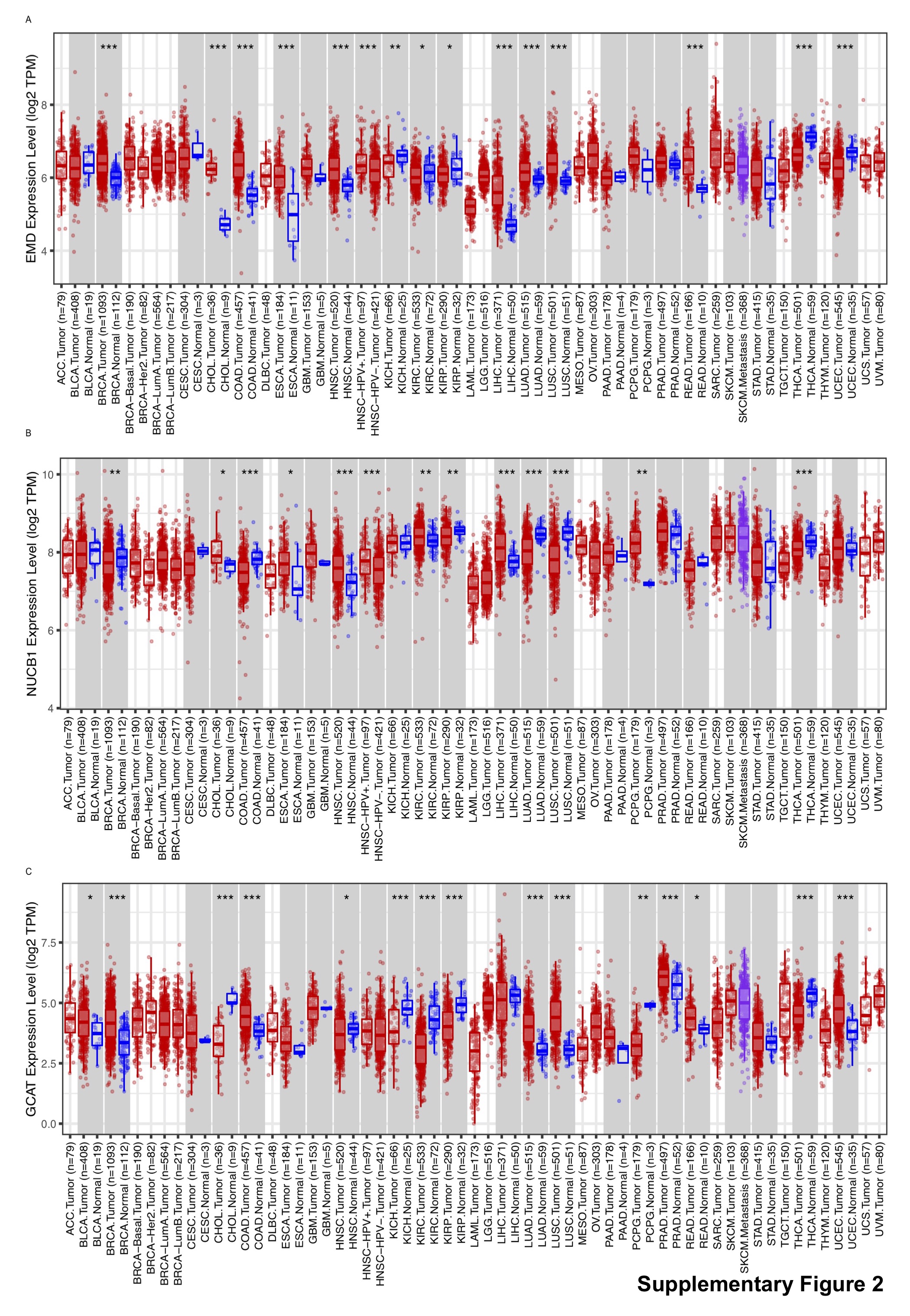

Supplement: Supplementary file 1 [file DataSheet1.ZIP › SI/Supplementary Figure 2. The mRNA expression of EMD, NUCB1, and GCAT in pan-cancer obtained from TIMER2.0 database.jpg]

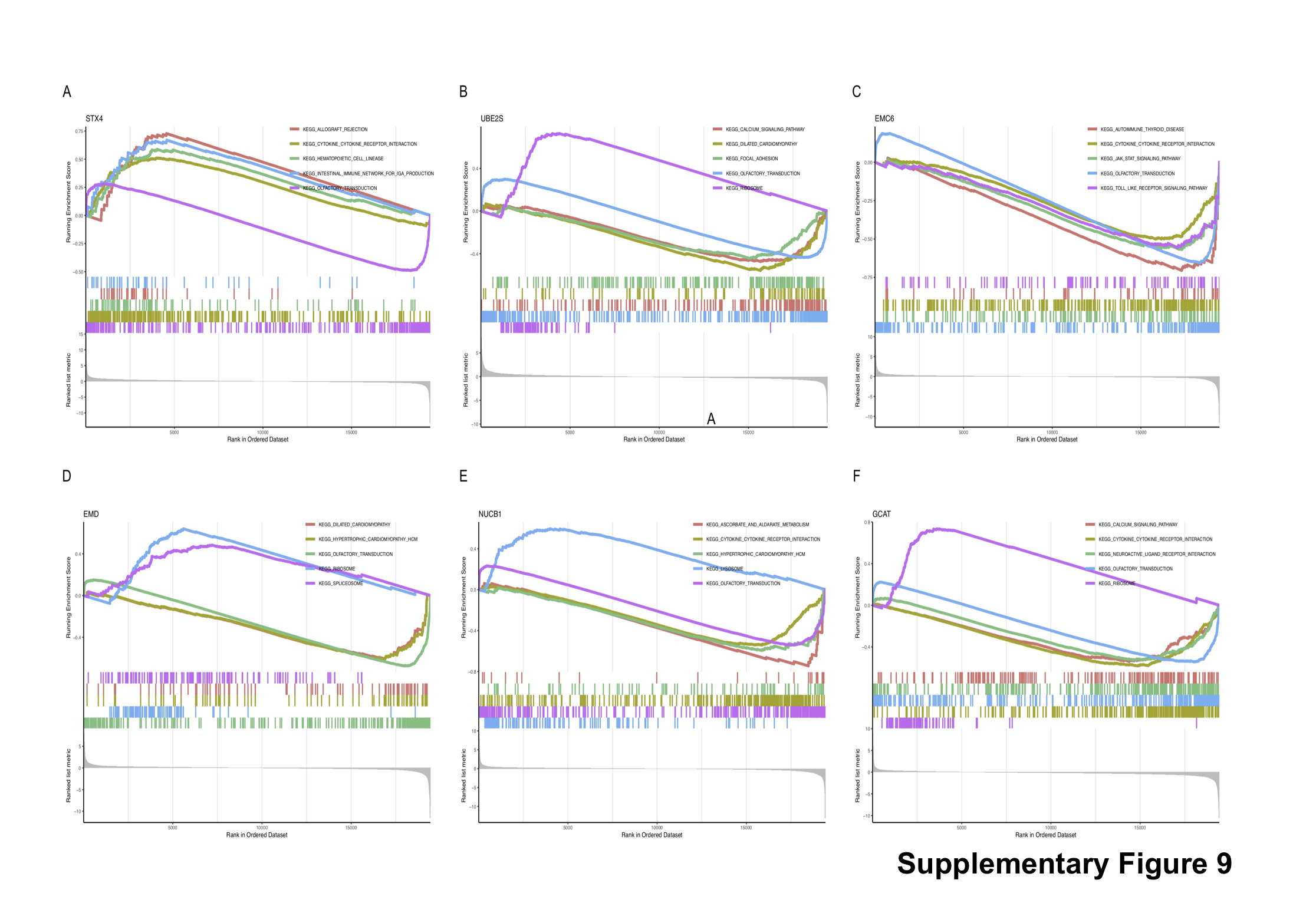

Supplement: Supplementary file 1 [file DataSheet1.ZIP › SI/Supplementary Figure 9. The enrichment pathways of GSEA in KEGG collection of TCGA-PRAD.tiff]

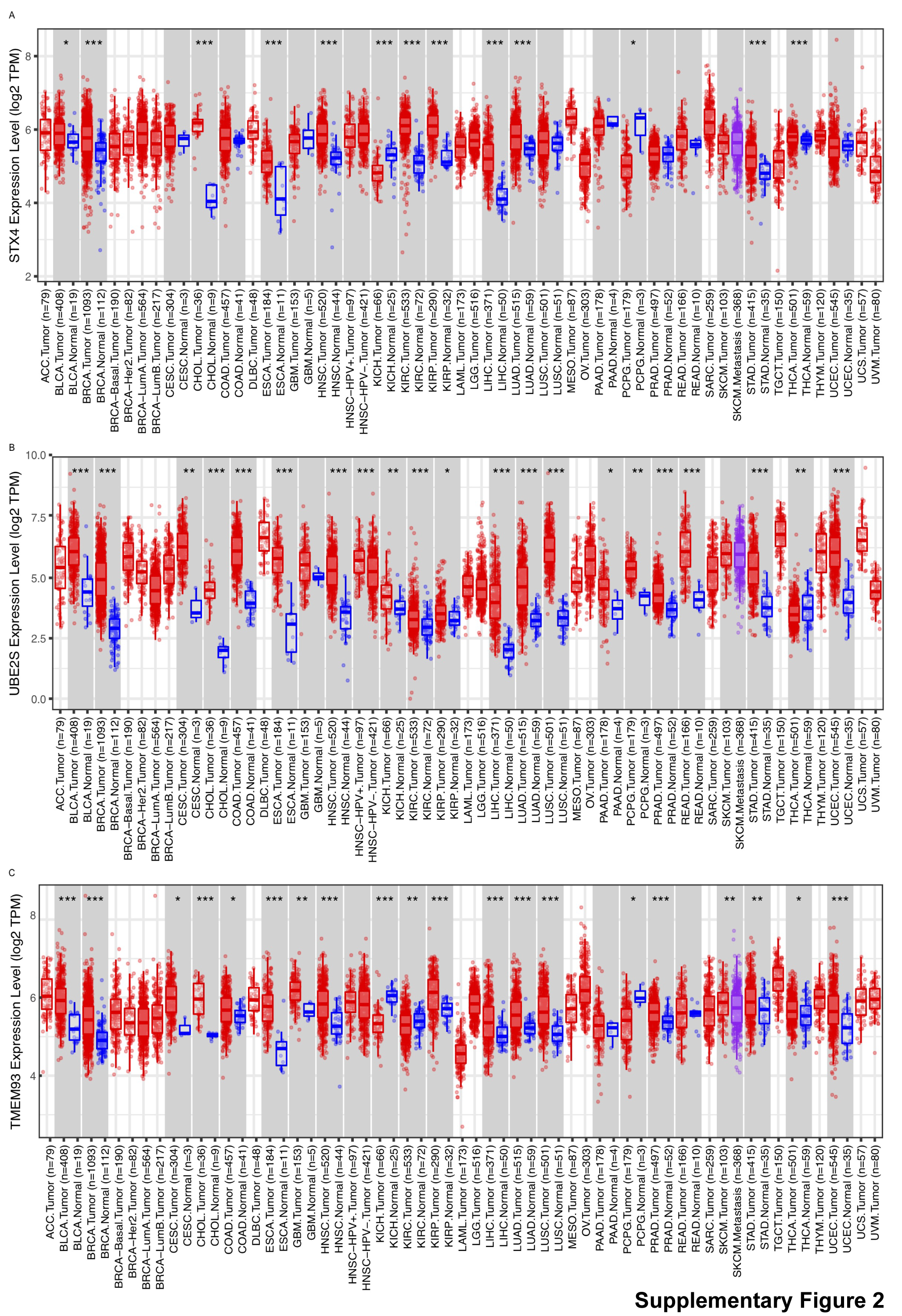

Supplement: Supplementary file 1 [file DataSheet1.ZIP › SI/Supplementary Figure 2. The mRNA expression of STX4, UBE2S, and TMEM93 in pan-cancer obtained from TIMER2.0 database.tif]

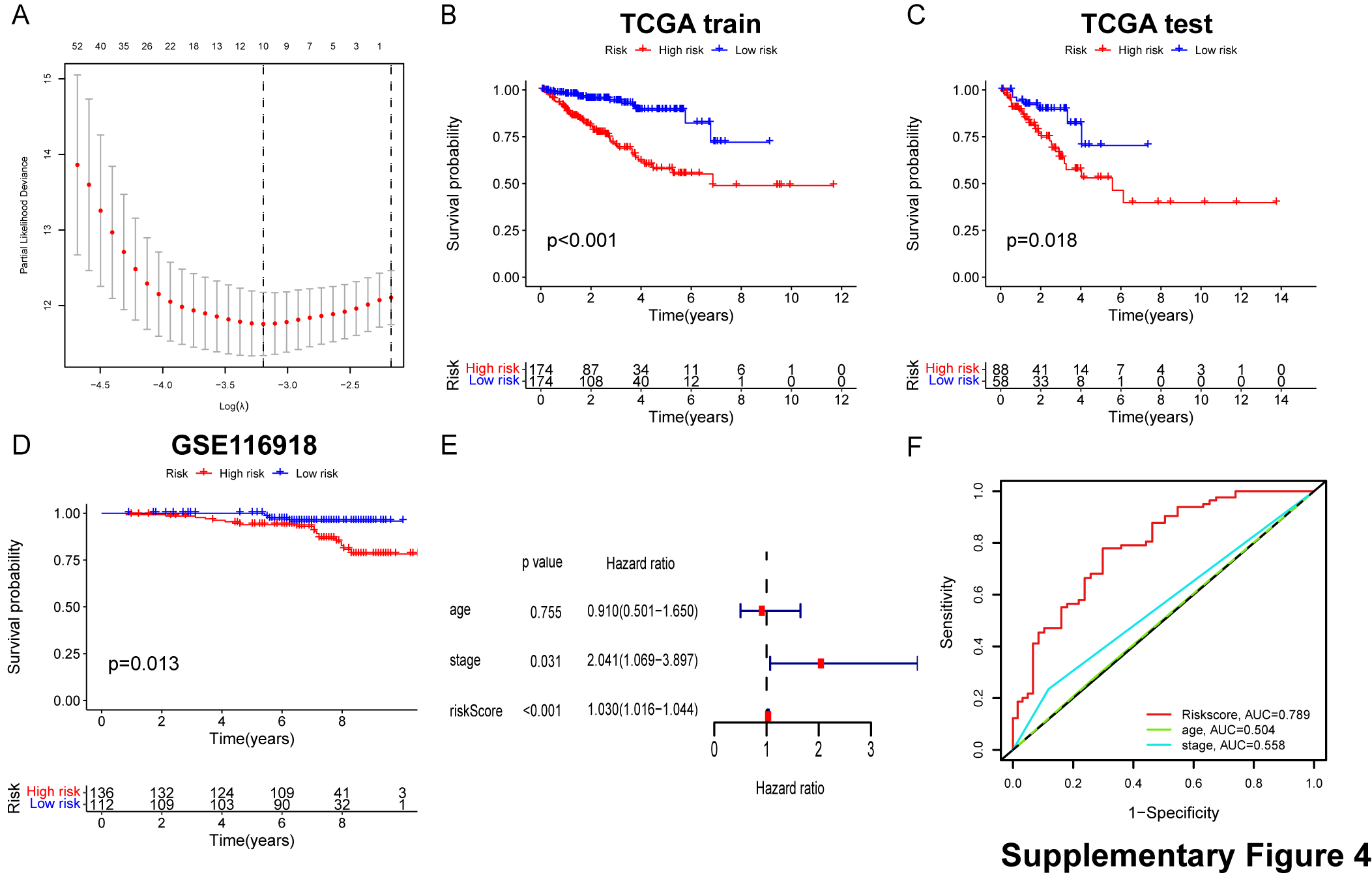

Supplement: Supplementary file 1 [file DataSheet1.ZIP › SI/Supplementary Figure 4. Establishment of the prognostic risk signature.tif]

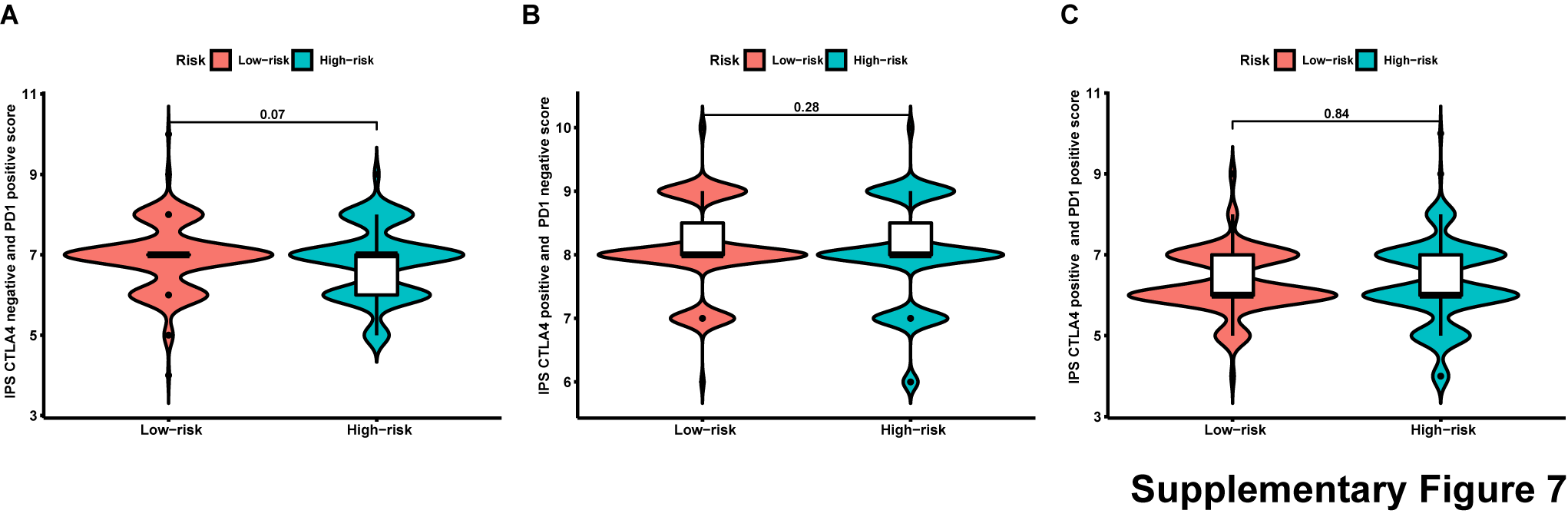

Supplement: Supplementary file 1 [file DataSheet1.ZIP › SI/Supplementary Figure 7. Correlation between risk score and immune checkpoint blockade therapy effect in PCa.tif]

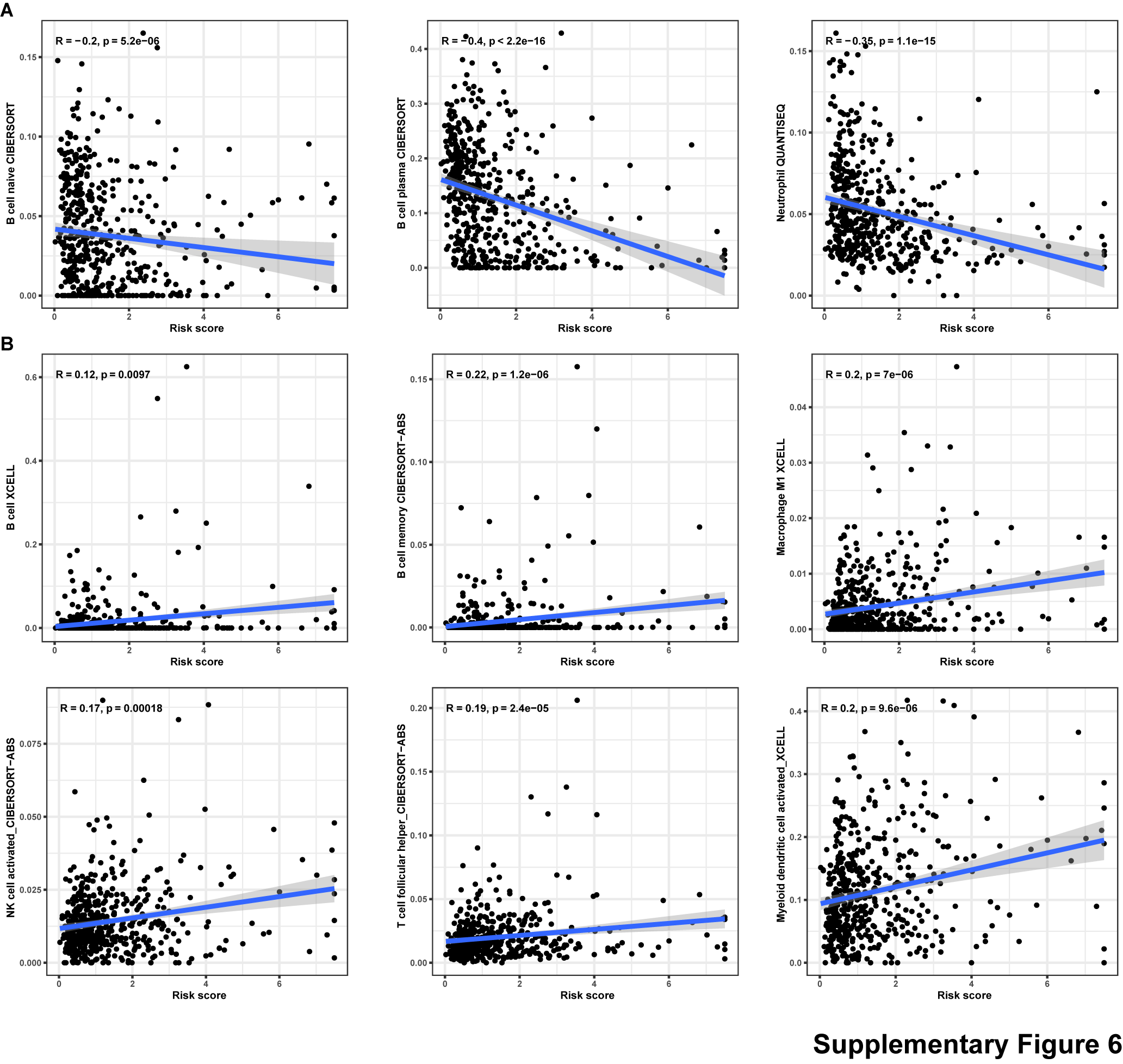

Supplement: Supplementary file 1 [file DataSheet1.ZIP › SI/Supplementary Figure 6. The correlation between the risk score and immune cell infiltration.tif]

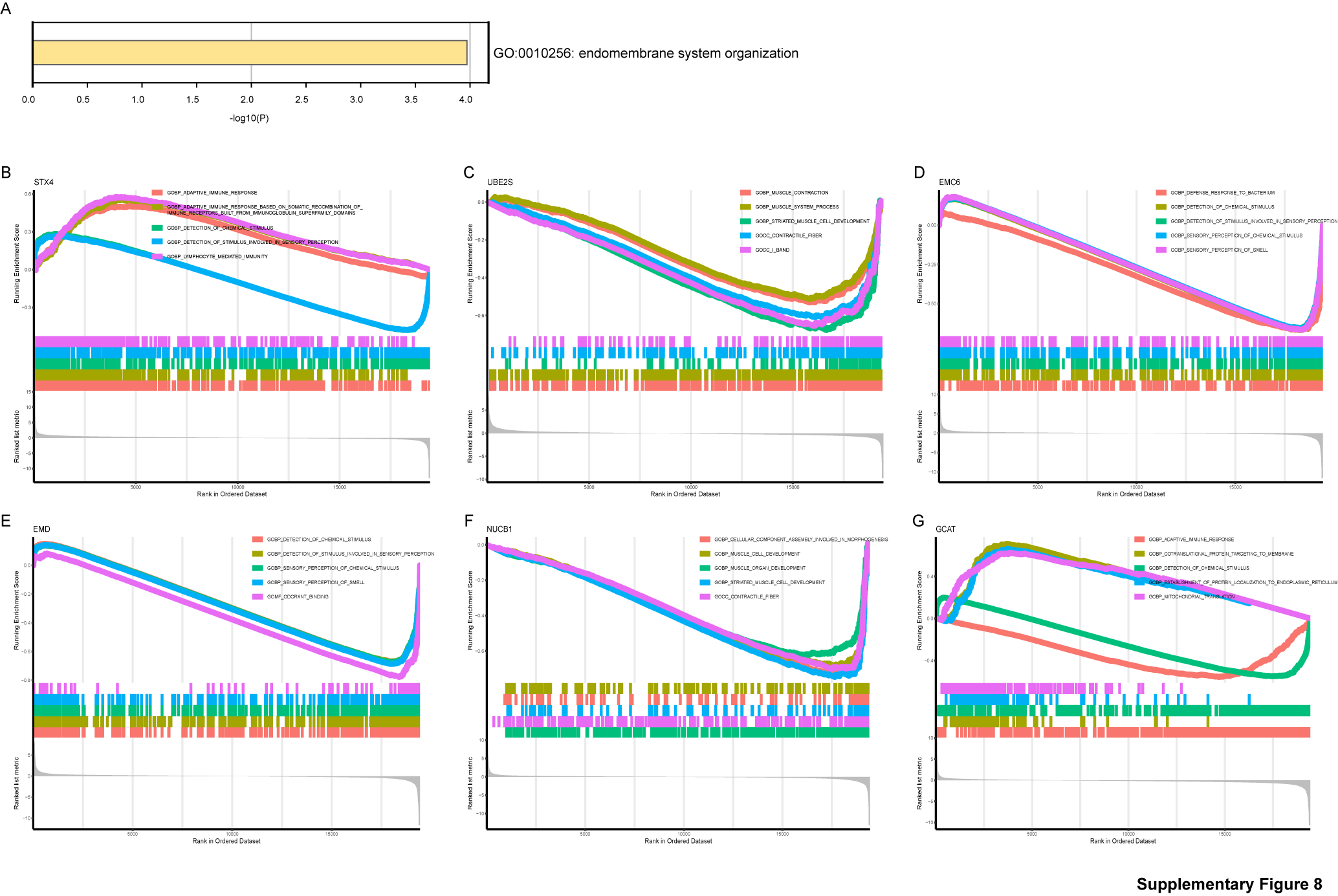

Supplement: Supplementary file 1 [file DataSheet1.ZIP › SI/Supplementary Figure 8. The enrichment pathways of GSEA in GO collection of TCGA-PRAD.tif]
